# Cancer Metastasis: The Role of the Extracellular Matrix and the Heparan Sulfate Proteoglycan Perlecan

**DOI:** 10.3389/fonc.2019.01482

**Published:** 2020-01-17

**Authors:** Zehra Elgundi, Michael Papanicolaou, Gretel Major, Thomas R. Cox, James Melrose, John M. Whitelock, Brooke L. Farrugia

**Affiliations:** ^1^Graduate School of Biomedical Engineering, UNSW Sydney, Sydney, NSW, Australia; ^2^The Garvan Institute of Medical Research and The Kinghorn Cancer Centre, UNSW Sydney, Darlinghurst, NSW, Australia; ^3^School of Life Sciences, University of Technology Sydney, Sydney, NSW, Australia; ^4^St Vincent's Clinical School, Faculty of Medicine, UNSW Sydney, Sydney, NSW, Australia; ^5^Raymond Purves Bone and Joint Research Laboratories, Kolling Institute of Medical Research, Royal North Shore Hospital, University of Sydney, St Leonards, NSW, Australia; ^6^Department of Biomedical Engineering, Melbourne School of Engineering, The University of Melbourne, Melbourne, VIC, Australia

**Keywords:** cancer metastasis, heparan sulfate proteoglycan, perlecan, heparanase, therapeutic

## Abstract

Cancer metastasis is the dissemination of tumor cells to new sites, resulting in the formation of secondary tumors. This process is complex and is spatially and temporally regulated by intrinsic and extrinsic factors. One important extrinsic factor is the extracellular matrix, the non-cellular component of tissues. Heparan sulfate proteoglycans (HSPGs) are constituents of the extracellular matrix, and through their heparan sulfate chains and protein core, modulate multiple events that occur during the metastatic cascade. This review will provide an overview of the role of the extracellular matrix in the events that occur during cancer metastasis, primarily focusing on perlecan. Perlecan, a basement membrane HSPG is a key component of the vascular extracellular matrix and is commonly associated with events that occur during the metastatic cascade. Its contradictory role in these events will be discussed and we will highlight the recent advances in cancer therapies that target HSPGs and their modifying enzymes.

## Cancer Metastasis

Metastasis of a tumor is the systemic dissemination and colonization of tumor cells from the primary tumor to a secondary site and is a major cause of cancer-related deaths (1). Cancer is a global epidemic with an estimated 18.1 million new cases and 9.6 million deaths occurring in 2018 (2). Metastasis is an inherently inefficient process, that involves spatial and temporal regulation by both intrinsic and extrinsic factors. It is generally assumed that a cancer cell's genetic mutational burden compounds with advancing malignancy, resulting in the acquisition of proliferative and invasive traits, and finally the capacity to metastasize and colonize, distant organs. However, mutational burden alone does not fully explain the capacity of cells to invade, disseminate, and metastasize to secondary sites (3–6). The role of the microenvironment is now becoming appreciated as a key element in cancer progression, which is driven by interactions between tumor cells and their microenvironment (7–9).

The extracellular matrix (ECM) is a non-cellular meshwork of crosslinked macromolecules including collagens, proteoglycans, and glycoproteins, that form a dynamic, supramolecular, scaffold. It provides cues, both physical and chemical, which influence cancer progression and metastasis. Biochemical and biomechanical cues present in the ECM, such as sequestered growth factors, ECM biomechanics and ultrastructural organization, are sensed by cells and converted into downstream cellular responses. These downstream cellular responses act in concert to alter malignant progression. Modulation of ECM components, by way of disrupted turnover, and aberrant or absence of post-translational modification (10), are some of the changes common to many diseases, including cancer (11, 12). Moreover, the ECM is a highly ordered structure, and its functional properties are contingent upon the precise assembly of ECM components (13). Subtle changes in the stoichiometry of these components may have downstream biological ramifications which affect tissue function. Cancer associated fibroblasts (CAFs) are important stromal cells within the tumor microenvironment that can be educated and/or recruited by tumor secreted factors. The capacity of CAFs to synthesize and remodel ECM components critically effects tumor progression (14). Understanding the nature of the heterotypic interactions between tumor cells, the ECM, and CAFs within the tumor microenvironment will offer insights into the mechanisms underpinning tumor progression and metastasis.

**Figure 1:**
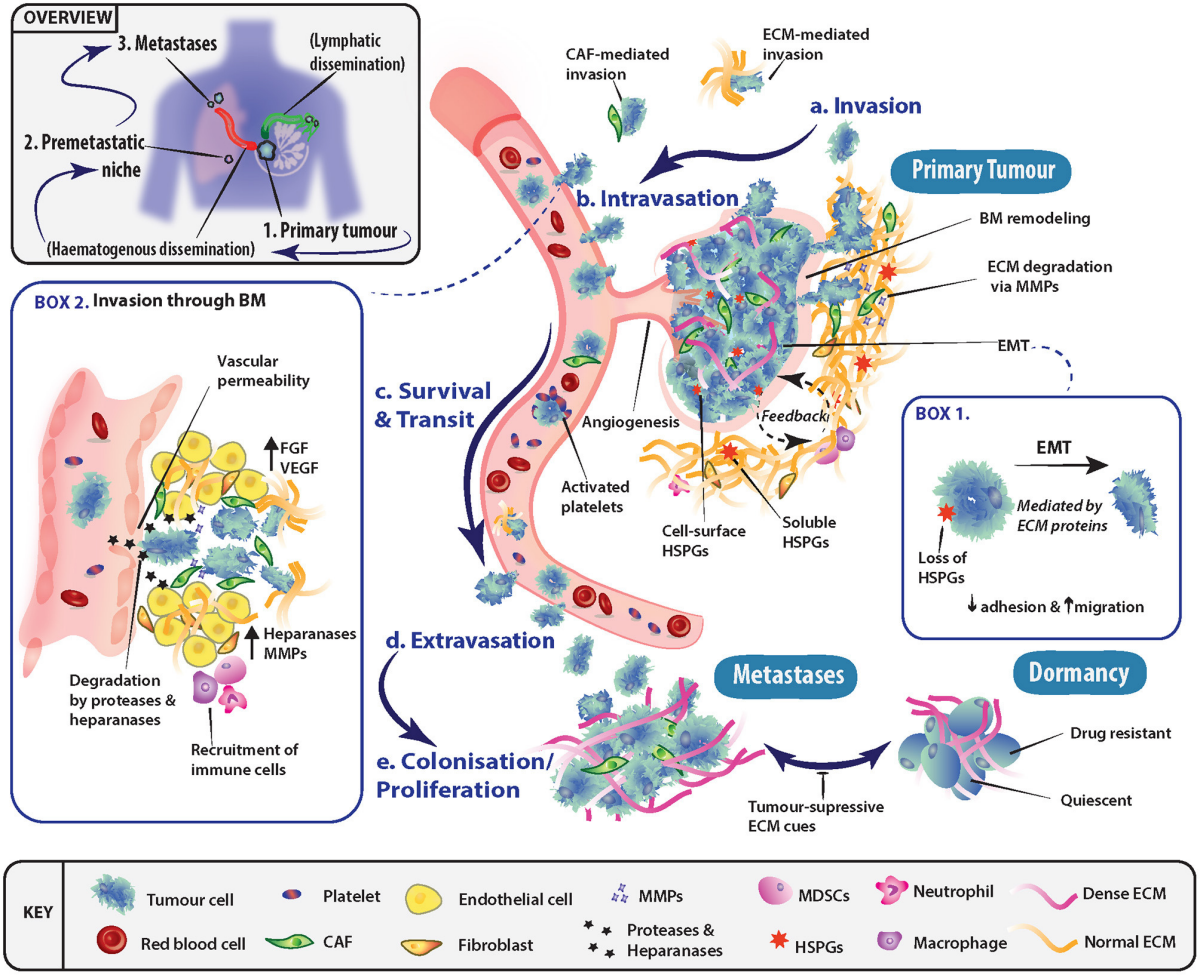
Role of the extracellular matrix in driving progression through stages of the metastatic cascade. **(a)** Primary tumor cells may undergo epithelial-mesenchymal transition (EMT) (Box 1) and invade through basement membranes (BM) into the surrounding stroma. Tumor cell local invasion and metastatic dissemination is often facilitated by cancer-associated fibroblasts (CAFs) or specific ECM components, which may enhance invasion or modulate the immune system. **(b)** To disseminate to a secondary site, tumor cells must access the vascular system and intravasate through the endothelial BM. This occurs in part through the release of proteases and heparanase, which disrupt BM integrity (Box 2). **(c)** The circulating tumor cells (CTCs) must then survive transit to secondary sites of metastasis and can be assisted by platelet activation as well as accompanying CAFs. **(d)** To exit the vessel, cells extravasate into the surrounding tissue and seed at distinctly different tissues from the primary tumor. Overt colonization of secondary sites by disseminating tumor cells (DTCs) is greatly enhanced through extravasation at premetastatic niches. **(e)** Extravasated cancer cells typically have three fates, either colonize and proliferate to form overt metastases, enter a reversible state of dormancy or, in most cases, die.

The process of metastasis is typically represented as a series of interconnected, and overlapping events, whereby certain conditions must be met before tumor cells transition to the next stage (Figure 1). These events include invasion into adjacent tissue, intravasation into the bloodstream and lymphatics, cancer cell survival during transit and extravasation out of vessels, and finally secondary organ colonization. The ECM is a key component throughout this cascade of events, with its involvement in modulating the behavior of both tumor and non-malignant stromal cells at all steps along the metastatic cascade.

### Epithelial-Mesenchymal Transition

Epithelial-mesenchymal transition (EMT) is one of the key programs in cancer that is thought to facilitate the shift in tumor cell behavior from a static epithelial phenotype to a more migratory, invasive, and mesenchymal one (Figure 1, Box 1). EMT and its regulatory signaling pathways are influenced by biochemical cues within the ECM. For instance, ECM environments rich in the glycosaminoglycan (GAG) hyaluronan (HA), transduce signals through the membrane receptor CD44, triggering EMT (15–18). The glycoprotein tenascin C has also been shown to be elevated in late stage mammary invasive ductal carcinomas at the tumor-stromal border. Here, it induces EMT through the proto-oncogene tyrosine-protein kinase Src and focal adhesion kinase (FAK) axis (19, 20). Furthermore, the shift in expression of the heparan sulfate proteoglycan (HSPG) syndecan-1 from tumor cell expression to stromal cell expression (*viz*. vimentin positive CAFs) has been shown to feed back onto cancer cells and drive EMT in many solid tumors (21). However, in contrast, Shen et al. (22) demonstrated that tubulointerstitial nephritis antigen-like 1 (TINAGL1), an ECM protein which competitively binds to integrins α5β1, αvβ1, and epidermal growth factor receptor (EGFR), can inhibit fibronectin-mediated FAK/EGFR signaling. This highlights how the balance between multiple ECM molecules can regulate the same intracellular signaling networks.

### Invasion and Intravasation

Tumor cell invasion is initiated through the breakdown of the interactions (i.e., cell-cell and cell-ECM) at the primary tumor site, allowing cells to invade into the adjacent tissue (Figure 1a), in conjunction with local remodeling of the adjacent basement membrane (BM). As tumor cells pass through the local microenvironment of the primary site, they are exposed to a milieu of biomechanical cues within the ECM such as tissue stiffness, density and porosity (23–25), which regulate tumor cell fate. Seminal work demonstrated the ECM's importance at initial stages of metastasis, where interactions between tumor cells and a fibrotic and stiff extracellular matrix induced a malignant and invasive phenotype, which could be blocked to re-establish tissue order (26). At the tissue organizational level, the alignment of collagen fibers has been shown to have prognostic value in breast cancer whereby collagen fibers aligned perpendicular to the tumor periphery, known as tumor-associated collagen signature-3 (TACS-3), are prognostic of patient survival (27, 28).

Hydration of tumor tissue is strongly influenced by the presence of specific glycosaminoglycans (GAGs) within the tissue, due to their anionic structure and their ability to attract water. As hydration increases, increased intra-tumoral hydrostatic pressure rises and alters the biomechanical properties of the tissue which is known to be crucial to invasiveness (29, 30). Perfusion of nutrients, growth and chemotactic factors are also affected leading to changes in cancer cell invasion (31). Finally, matrix metalloproteinases (MMPs) released from both tumor and stromal cells degrade the ECM and facilitate local invasion (32, 33). The release and activation of ECM-sequestered growth factors [e.g., transforming growth factor (TGF)-β, fibroblast growth factors (FGFs)] may also play a part in this malignant process (34).

Following local invasion at the primary site, tumor cells typically spread around the body via the hematogenous or lymphatic networks which requires traversing the vascular and/or lymphatic BMs (Figure 1b, Box 2). However, tumors need not be clinically advanced for this to occur, as dissemination has been observed very early in tumor formation, even before clinical symptoms of disease are evident (35, 36). BMs are specialized tissues underlying epithelial and endothelial structures. BMs are membrane like structures with low porosity and their constituents are densely arranged together. Thus, for cells to traverse BMs, known as intravasation, they require the activation of specific protease-dependent and -independent programs (37–39). BMs impart polarity and survival signals to cells in contact with them, in addition to acting like a molecular sieve for the perfusion of nutrients and molecules from the blood through to the interstices. As such, the structural integrity of vessels and their BMs presents a major obstacle to invading tumor cells. However, in cancer, disruption of BMs is commonly observed. A series of recent studies (40, 41) demonstrated that the ECM molecule hyaluronan and proteoglycan link protein-1 (HAPLN1) decreased with aging of the ECM. This resulted in disruption of the vascular BM and increased vessel permeability, leading to subsequently enhanced melanoma metastasis in mice. In addition, HA has been shown to be important in the regulation of vascular endothelial barrier permeability, through stabilization of cell-cell junctions (42, 43). Furthermore, high molecular weight HA secreted by tumors has been shown to negatively regulate hyaluronan binding protein 2 (HABP2), a serine protease, which is known to compromise vessel integrity (44). Along with the release of proteases by tumor cells, invasion through BMs can be affected by the release of heparanase (45, 46), which degrades the HS chains of HSPGs located in the BM and ECM, as reviewed by (47) (Figure 1, Box 2).

### Survival and Transit Through the Circulatory System

Once tumor cells enter the circulation, their survival in the absence of cell-cell and cell-ECM cues is a crucial factor determining metastatic outcome (Figure 1c). Various mechanisms have been uncovered which facilitate cancer cell survival in the circulation. For example, circulating tumor cell (CTC) clusters exploit mechanisms such as tropomyosin receptor kinase B (TrkB) signaling to combat apoptosis induced by the lack of cell-ECM interactions, termed “anoikis” (48, 49). In addition, the close association of stromal elements with tumor cells in circulation, namely CAFs and their secreted factors (e.g., FGFs) enhance survival and facilitate metastasis (50). Platelet derived TGF-β signaling also protects against the lack of cell-ECM interactions present in circulation, through inducing a mesenchymal-like phenotype (51). The activation of platelets provides CTCs with fibrinogen (52) and tissue factor (53), which protects against immune clearance within the circulation and at secondary sites. The cues provided may temporarily be substituting for the absence of correct tissue and ECM contacts, and therefore likely provide survival signals that protect cancer cells (7).

### Extravasation

Tumor cells that survive within the circulation and lodge in the vasculature of secondary organs, must extravasate into the parenchyma in order to begin the colonization process (Figure 1d). The site of extravasation may be determined to some extent by the formation of “pre-metastatic niches” (54), which can in part explain metastatic organotropism (55). Of note, secreted factors from the primary tumor, such as MMP-3, -9 and -10 (56, 57), can induce the production of vessel destabilizing factors at secondary sites of future metastasis, which act to enhance extravasation. Once extravasated into secondary organs, tumor cells must adapt to the new local cues (i.e., ECM molecules as well as locally secreted growth factors) in order to persist and go on to form overt metastases (Figure 1e). At this stage, the alternatives are entry into a dormant state, or ultimately death. Therefore, this phase in the cascade relies on the interaction between the extravasated tumor cells and the characteristics of the host tissue microenvironment for the successful establishment and outgrowth of overt metastases.

### Secondary Organ Colonization

More recently, it has become increasingly apparent that secondary sites may not simply be naïve recipients of disseminated cells, and instead, the ECM and local microenvironment may be remodeled prior to the arrival of tumor cells. This concept has been termed the pre-metastatic niche (54, 58–60), and encompasses the idea that primary tumors were capable of remodeling the tissue microenvironment of secondary organs prior to their arrival in order to facilitate metastatic colonization (Figure 1). This was first demonstrated by Kaplan et al. (60) who showed that bone marrow derived hematopoietic progenitor cells, activated by secreted factors from the primary tumor, are capable of remodeling secondary lung tissue to produce a fibronectin-rich environment prior to tumor cell arrival. This environment then acts to support overt colonization by the seeding tumor cells. Cell-ECM interactions not only supply an anchorage point for seeding, but also activate survival and proliferative signaling programs transduced through integrin complexes and their associated downstream signaling (61–63). These cell-ECM interactions, and signaling networks are potential targets for therapeutic intervention, such as has recently been shown for ROCK inhibition (64, 65). CTCs arriving in secondary organs typically initiate and drive ECM remodeling at these sites. For example, breast cancer cells metastasizing to the lung produce their own tenascin C that promotes survival and macrometastatic outgrowth via NOTCH and WNT stem cell pathways (66). This is further perpetuated by secretion of TGF-β by cancer cells, which stimulates fibroblasts to secrete periostin (POSTN), further activating WNT signaling (67). Additionally, when secreted at elevated levels, bone morphogenic protein (BMP)-4 and -7 have been demonstrated to cause cancer cell dormancy in both lung (68, 69) and bone (70), which is driven by secreted protein acidic and rich in cysteine (SPARC) in the prostate cancer setting (71).

Another example of ECM induced dormancy has been observed within the “perivascular niche,” which, in some tissues, such as bone and lung, produce a source of quiescing thrombospondin 1 (TSP1) (72). Upon vascular disruption, in situations such as inflammation or wounding, TSP1 secretion is disrupted and the generation of a tumor-promoting microenvironment ensues and facilitates metastatic outgrowth (72–74). Additionally, vascular endothelial cell secretion of perlecan has also been shown to influence lung cancer cell dormancy in the perivascular niche (75). Perlecan has also recently been shown to be upregulated in CAFs in pancreatic cancer through secretion of TNFα from p53 gain-of-function (but not p53 loss-of-function) cancer cells. Cancer cell education of CAFs and the elevated secretion of perlecan was responsible for the generation of a prometastatic microenvironment (76).

It is clear that the ECM is a key regulatory determinant of tumor cell phenotype and behavior, which is dynamically modified throughout the different stages of metastatic progression. The inherent nature of a patient's ECM and the particular modifications accrued by the ECM throughout tumorigenesis may be viewed as either necessary and/or sufficient to enable malignant progression. Thus, the tumor ECM represents a vast territory of underexploited therapeutic targets in treating cancer and cancer metastasis.

## Proteoglycans and Their Glycosaminoglycan Chains

Glycosaminoglycans (GAGs) are well-established regulators in the metastatic spread of cancer (77–82). GAGs are negatively charged glycan structures comprised of repeat disaccharide units and belong to one of four subgroups: (1) heparin/HS, (2) chondroitin/dermatan sulfate (CS/DS), (3) keratan sulfate, and (4) hyaluronic acid or HA. All GAGs, other than HA, are covalently attached to the core protein of proteoglycans (PGs). HSPGs are ubiquitously expressed and consist of a protein core to which HS chains are covalently linked. Biological activities associated to HSPGs are mediated through interactions with various ligands, via the protein core or the HS side chains, where the specificity and affinity of these interactions is related to the HS chain structure and position of sulfate groups (83, 84). HSPGs are involved in multiple roles ranging from structural development and maintenance, to organization of the ECM and BM via binding with matrix molecules including collagen IV, fibronectin, and laminin (85, 86). In particular, HS modulates cell-cell interactions by acting as a co-receptor for different cell surface receptors as well as influencing cell-ECM interactions. HS also mediates the sequestering of various growth factors, chemokines, cytokines, morphogens, and enzymes by forming protected “reservoirs” that upon release can promote receptor-ligand signaling complexes to mediate crucial regulatory roles in cellular processes to maintain tissue homeostasis (87). Structural modification of HS can occur post-translationally by the actions of sulfotransferases, sulfatases (Sulfs), heparanase. MMPs and other proteolytic enzymes (e.g., plasminogen) can modify the protein core of HSPGs and can therefore regulate HSPG-dependent signaling pathways (88, 89). Heparanase is the only mammalian derived enzyme that is capable of degrading HS (90) as well as heparin (91). HSPGs regulate a myriad of activities including; cell adhesion and migration, proliferation, differentiation and morphogenesis, vascularization, cytoskeletal organization, and tissue repair (92). These phenomena are essential for metastasis onset and success.

### Heparan Sulfate Proteoglycans

HSPGs have intracellular, cell surface, and ECM localizations, including the BM (93). The BM PGs, perlecan, agrin, and collagen XVIII are primarily substituted with HS GAGs. Endothelial, epithelial, immune cells, and fibroblasts all synthesize these HSPGs, though HSPGs produced by different cell types will be decorated with HS chains that differ in structure, and thus their biological interactions will also differ (94). Hence, HSPGs have been reported to have both pro-angiogenic and anti-angiogenic properties due to heterogeneous HS structures and thus, their interactions with numerous growth factors differ (95). Cell surface HSPGs belong to members of the transmembrane syndecan (SDC) and the glycosylphosphatidyl-inositol (GPI)-anchored glypican (GPC) families. There are four mammalian SDCs (SDC 1-4) and six GPCs (GPC 1-6). The location of HS chains on the PG protein core with respect to the cell surface differs between SDCs and GPCs. The HS chains that decorate GPCs are located close to the plasma membrane. In the SDCs, the HS chains are located at sites further away from the cell surface. The SDC family members are differentially expressed on different cell types, SDC-1 is found on epithelial cells, SDC-2 on fibroblasts and endothelial cells, SDC-3 is on neural cells, and SDC-4 is ubiquitously produced by most cell types but in relatively low abundance (96). Shedding of cell surface HSPGs provides another mechanism to control HSPG distribution, as SDCs can be enzymatically released by MMPs, where GPCs are shed by GPI-specific lipases (97, 98). While, HSPG shedding downregulates their functions at the cell surface, the shed, and now soluble, HSPGs may facilitate the transfer of bound ligands to signaling receptors on neighboring cells conveying positive or negative effects in cancer progression (99). Opposing roles for anchored vs. shed GPCs have been demonstrated. Overexpression of GPC-3 in hepatocellular carcinoma (HCC) promotes tumor growth via WNT (100) and insulin-like growth factor (IGF) signaling (101). However, soluble GPC-3 blocks WNT signaling and inhibits HCC growth (102). Similarly, transmembrane GPC-1 promotes proliferation and metastatic growth of pancreatic cancer cells (103, 104), whereas, soluble GPC-1, inhibits the mitogenic response to FGF-2 and heparin-binding EGF-like growth factor (HBEGF) (104). Additionally, glycoproteins such as betaglycan and CD44v3 are part-time HSPGs, and may have potential roles in cancer (105, 106).

The strategic location of HSPGs in tissues are critical to their functional roles. Localization of SDCs and GPCs in the plasma membrane regulates intracellular and cell-ECM signaling. Localization of HSPGs in the BM regulates their barrier functions and co-ordinates cell-cell/ECM-cell interactions. Localization of perlecan at the interface of tissues and tissue layers, coupled with their sequestered growth factors, has been hypothesized as on-site “depots” that assist with the restoration of those borders when compromised (107). Cell surface HSPGs can also act as docking modules for MMPs (108, 109), which promote invadopodia and enable cells to move in specific directions through the ECM (110). MMPs secreted by invadopodia promote the invasion of breast carcinoma cells into the ECM (111). Endothelial cells also release granules containing MMP-2 and MMP-9 at focal sites, and their focal MMP activation can contribute to directed angiogenic events (112). It has been proposed that cell surface HSPGs generate a tract in the ECM for the migration of cells. Weak interactive properties between cells and HS allow the cell to “walk” along the cell surface or ECM HS chains facilitating cellular migration (108). Shed fragments of cell surface HSPGs can also influence cell proliferation by amassing in intracellular spaces and sequestering growth factors (86). Degradation of HS, by heparanase, on SDC-1 produces heparin-like fragments that activate FGF-2 mitogenicity (113). The biological role of a HSPG therefore depends on the properties of its protein core, the number of GAG chains attached, its localization in cells and tissues, as well as the biosynthetic modifications its GAG chains receive *in situ*.

**Figure 2:**
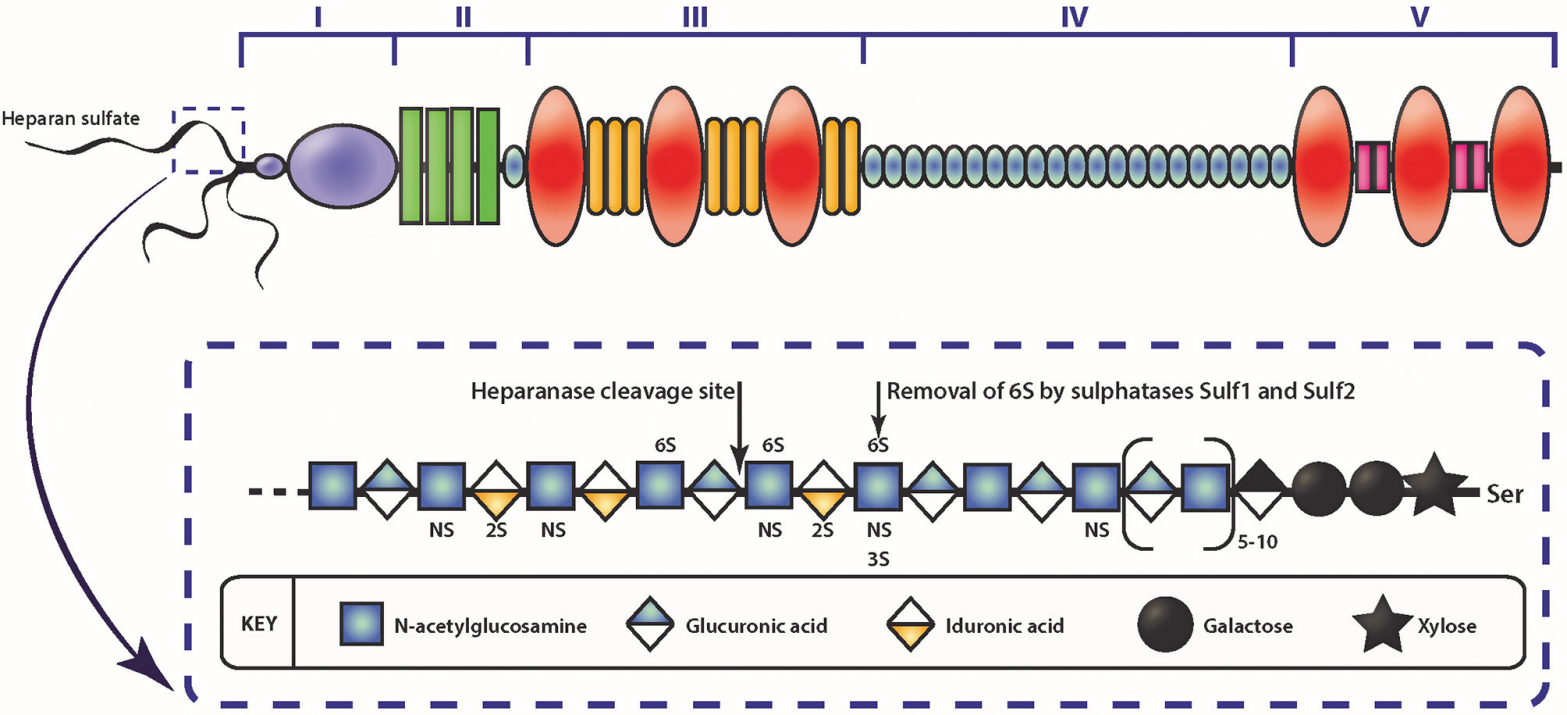
Schematic diagram of the HSPG perlecan and HS. The different domains of perlecan are depicted by roman numerals. The insert depicts a schematic of HS represented by the repeating disaccharide of N-acetyl glucosamine and glucuronic acid (or iduronic acid) and sulfate moieties that can occur. Enzymatic modification of HS can occur via heparanase cleavage, resulting in smaller molecular weight fragments, or cleaving the 6-O sulfate on glucosamine via sulfatase.

The vast range of biological functions attributed to GAGs in cancer metastasis, and numerous other biological events, is due to their non-templated controlled, highly heterogeneous and complex structure, which enables the regulation of tissue-specific functions. Biosynthesis of GAGs is a sequential process that occurs in the endoplasmic reticulum and the Golgi apparatus (114). This process is governed by a large family of enzymes, and while the function of these enzymes is known, the process that controls specific GAG structure, as well as the degree and position of sulfate motifs is not. HS, the major GAG discussed herein, consists of a glucuronic acid-galactose-galactose-xylose-linker region (GlcA-Gal-Gal-Xyl) which is initiated by the enzymatic transfer of xylose to specific serine-glycine residues of core protein sequences (115). HS assembly occurs by sequential addition of N-acetyl glucosamine (GlcNAc) to the linkage tetrasaccharide acceptor, then GlcA to form GlcA-GlcNAc disaccharide repeats (Figure 2). As the chain polymerizes, HS is also enzymatically modified by sulfotransferases and an epimerase at various positions in a coordinated manner, with the product of one modification serving as substrate for the next step (116). The enzyme, N-deacetylase/N-sulfotransferase (NDST), substitutes the N-acetyl group with a sulfate group in between clusters of GlcNAc, leaving regions of the chain unmodified. Further modifications include; epimerization of GlcA to iduronic acid (IdoA) and 2-O-sulfation of IdoA, O-sulfation of GlcNS by sulfotransferases at C6 or less commonly, at C3. Thus, sulfation along HS chains is not uniform and contains highly sulfated regions (NS domains) and largely unmodified regions (NA domains). Ligand binding to HS depends on the arrangements of NS and NA domains, and on the modified residues within the NS domains. The HS-FGF-2 interaction exemplifies a GAG-growth factor interaction and demonstrates how specific HS structures facilitate FGF-2/FGFR-mediated signaling. HSPGs play a vital role in the FGF-2/FGFR interactions by assembling FGF-2 near the receptor, which forms a ternary complex that stabilizes the ligand-receptor complex, thereby promoting signal transduction (117). HS chains require N-sulfated glucosamine and 2-O-sulfated IdoA units to bind to FGF-2 (118). At the same time, for HS chains to bind FGFR, they require 6-O-sulfated GlcN residues along with 2-O-sulfated IdoA with N-sulfated GlcN residues also reported to be involved in this interaction (119).

A number of studies have highlighted that HS dysregulation in cancer can occur when the expression and behavior of HS-synthesizing and HS-modifying enzymes are altered (120–124). For instance, Weyers et al. reported on the structural differences found in sulfation patterns between normal and breast cancer tissues in addition to differences in sulfation between patients with non-lethal and lethal cancer (121). Specifically, patients with lethal cancer presented with decreased levels of 6-O sulfation of HS, and increased levels of unsulfated disaccharides. Furthermore, observed increases in HS chain length suggested that the breast tissue underwent changes in the HS polymerization pathway. A similar study assessing transcriptional patterns in panels of breast, prostate, colon cell lines, and isolated tumors confirmed that changes in HS biosynthetic enzyme levels occurred in a tissue-specific manner and particularly affected modification enzymes which undertake HS sulfation (120); supporting previous studies in animal models (125, 126). Interestingly, the authors also discovered that there was no difference in the biosynthetic enzymes between normal and metastatic cell lines and proposed that the cells maintain relatively normal PG expression pattern at the cell surface in order to avoid immune detection.

The two known human orthologs of sulfatases (HSulf-1 and HSulf-2) are released as soluble enzymes capable of cleaving the 6-O sulfate on glucosamine (127). Despite similarities in their structural organization and mechanistic action, these sulfatases have been shown to have opposite roles in cancer progression. HSulf-1 suppresses FGF-2-mediated tumor cell proliferation and invasion, HSulf-2 augments these activities to progress disease, as examined in HCC (128). HSulf-1 is downregulated in breast, pancreatic, ovarian, head and neck cancers according to a tumor suppressor effect (129). HSulf-2 has additional roles in the pathogenesis of non-small-cell lung carcinoma (NSCLC), pancreatic cancer and glioblastoma despite unaltered expression levels (130, 131). In contrast, prostate cancer cells overexpressing HSulf-2 present with reduced levels of the trisulfated disaccharide UA(2S)-GlcNS(6S) in conjunction with an increase in EMT markers and WNT signaling (132). In this regard, the role of HS-modifying enzymes in regulating EMT is noteworthy, given its important role in metastatic progression (133, 134). For instance, Maupin et al. consistently found upregulation of the HSulf-2 enzyme in various *in vitro* models mimicking aspects of pancreatic cancer EMT (135). Furthermore, increased methylation of the HSulf-1 promotor was found to be present in samples from gastric cancer patients (55%) as compared to healthy patients (19%) (136). This was measured using cell-free serum samples taken from patients and the authors advised that methylation-induced silencing of HSulf-1 showed potential as an early diagnostic tool for cancer. Likewise, other studies have proposed that specific biosynthetic trends for each tumor type (121) or proteoglycan staining patterns based on associated GAGs could serve as potential prognostic biomarkers in various histological types (123). Certainly, this area of research will continue to evolve as new analysis tools become available to study GAG structure and identify key structure-function relationships. Significantly, tumor cells have been reported to actively manipulate the binding capacity of their HSPGs for FGF-2 and other growth factors, by modifying the overall density and sulfation pattern of their HSPGs (81). Since natural killer (NK) cells recognize particular HS fine structural patterns, explicitly 6-O-sulfonation and N-acetylation patterns, cancer cells can change their HS patterns to evade NK cells and immune surveillance (137, 138). Studies of breast and pancreatic cancer cells that express increased extracellular heparanase and aberrant HSulf activity have also been shown to affect recognition by NK cells (139).

### The Role of Perlecan in Cancer Metastasis

Among the various contributory factors so far identified to be involved in the various stages of cancer progression, perlecan, a modular HSPG stands out as an important player. Perlecan contains multiple domains (Figure 2) which allows participation in a variety of roles, as well as being a major structural constituent of BMs (85, 107, 140–143). Perlecan is encoded by the HGPS2 gene, and is predominately substituted with HS chains, though depending on the cell type it originates from, it may be substituted with CS, DS, a combination of HS, CS, and/or DS, or as a GAG-free glycoprotein (144, 145). The N-terminal Domain I is most commonly decorated with three HS chains, whereas at the C-terminal, Domain V can also be substituted with HS and/or CS chains (146). The protein core is divided into five domains, with each domain involved in binding to various partners, from classical ECM components such as collagen IV, nidogen-1, and fibronectin, to growth factors, including FGF-2, -7, vascular endothelial growth factor (VEGF) and platelet derived growth factor (PDGF) (85, 147, 148). While it is present in the BM of most endothelial and epithelial cells, perlecan also associates with the cell surface via interaction with α2β1 integrin (149). The c-terminal fragment of perlecan can exist as a separate fragment to the perlecan protein core, known as endorepellin, though it is not separately synthesized but rather is a result of proteolytic cleavage of secreted perlecan by proteases (150).

Interestingly, the two other HSPGs of BMs, agrin, and collagen XVIII, do not share much structural homology with perlecan, with the exception of Domain V of agrin (142). Although Domain I is unique to perlecan (151), it does contain the SEA (Sperm protein, Enterokinase, Agrin) module, which is present within other ECM proteins. GAG decoration on perlecan has been shown to be modulated by the presence of the SEA module since its deletion results in a recombinant protein with decreased HS content and an increase in CS (152). The importance of GAG decoration on perlecan has been further demonstrated in Hspg2^Δ3/Δ3^ mice, whereby deletion of exon 3 of the Hspg2 gene removes the GAG attachment sites in Domain I and the mice presented with impaired angiogenesis, delayed wound healing, and retarded tumor growth (153). The functions that perlecan Domain I plays in various cellular functions cannot be overstated, most notably in angiogenesis (141–143, 154) and is predominantly due to the GAG chains that decorate this domain. The HS moieties of perlecan can bind a variety of pro-angiogenic factors including FGF-1, -2, -4, -7, -10, hepatocyte growth factor and TGF-β (85, 142, 154, 155). The pro-angiogenic activity of perlecan is achieved primarily through the interaction between HS, that decorate the protein core, FGF, and its corresponding receptors. These interactions actively coordinate cell proliferation, motility and adhesion (94, 156, 157). Conversely, and despite being a key region within a pro-angiogenic parent molecule, endorepellin is a potent inhibitor of angiogenesis (158, 159). Endorepellin, via the protein core, binds to both VEGFR-2 and α2β1 on endothelial cells triggering a signaling cascade that disrupts cell actin cytoskeleton and inhibits cell motility (149, 158, 160). Endorepellin is also reported to have transcriptional control by suppressing HIF-1α, a key transcription factor involved in promoting angiogenesis (159). Endorepellin is comprised of three laminin-like globular domains (LG1-LG3) with most of the biological activity attributed to LG3, cleaved from the parent molecule by protease digestion (161, 162). Circulating LG3 levels have been shown to be reduced in breast cancer patients and are being explored as a biomarker for cancer progression and invasion (163). The expression of perlecan has been investigated in various cancer types both *in vitro* and *in vivo* (Table 1). Although the findings are inconsistent, it is apparent that perlecan controls cancer progression by regulating interactions between cells and signaling molecules during the various stages, including ECM dysregulation, angiogenesis and invasion, which will be discussed in the following sections.

**Table 1:** Summary of *in vivo* observations for perlecan expression in various cancer types.

**Cancer type**	**Assessment technique**	**Observations**	**References**
Melanoma	Immunohistochemistry	Increased in BM at tumor-stroma interface and surrounding blood vessels Increased levels in tissue	(164)
	mRNA expression	Increased levels in tissue	(165)
Colon	Immunohistochemistry	Increased in stroma	(166)
Lung	Immunohistochemistry	Decreased to undetected in BM at tumor-stroma interface	(167)
	mRNA expression	Increased levels in tissue	(165)
Breast	Immunohistochemistry	Decreased to undetected in BM at tumor-stroma interface	(168, 169)
	mRNA expression (*in situ*)	Increased levels in tumor and stromal cells	
	Immunohistochemistry	Increased in stroma	(166)
Heptocellular carcinoma (HCC)	Immunohistochemistry	Increased in BM at tumor-stroma interface and blood vessels in stroma	(170)
	Immunoelectron microscopy	Increased at BM at tumor-stroma interface	
Intraheptatic cholangiocarcinoma (ICC)	Immunohistochemistry	Decreased to undetected in stroma	(171)
	mRNA expression (*in situ*)	Increased levels in tumor cells and stromal fibroblasts	
Ameloblastoma	mRNA expression (*in situ*)	Increased levels in stromal cells	(172)
Prostate	Immunohistochemistry	Increased in stromal cells	(173)
Ovarian	Immunohistochemistry	Decreased to undetected in BM at tumor-stroma interface Unaltered in BM of surrounding blood vessels or stroma	(174)
Pancreatic	Immunohistochemistry	Increased in BM and stroma	(175)
Oral squamous cell carcinoma (SCC)	Immunohistochemistry	Decreased to undetected in BM at tumor-stroma interface Increased in stroma	(176)
Glioblastoma	mRNA expression	Increased levels in tissue	(177)

#### Extracellular Matrix Dysregulation

Cells interact with the ECM to regulate their activities and behavior. This interaction can occur directly through cell surface receptors, including integrins and discoidin domain receptors, and indirectly, via the release of growth factors and cytokines sequestered in the GAG chains (88, 178). ECM remodeling is instrumental to these essential functions including a fundamental role in angiogenesis (179). ECM remodeling removes the restrictive physical barrier, liberating endothelial cells to proliferate and migrate, which is coupled with the release of sequestered pro-angiogenic growth factors from HS chains of perlecan. The ECM is constantly deposited, remodeled, and degraded during development through to maturity to maintain tissue homeostasis (180, 181). Tissue inhibitor of metalloproteinase 3 (TIMP-3) inhibits ECM turnover and has been associated with cancer (182). This enzyme binds to sulphated GAGs on perlecan; further highlighting the significance of sulfation patterns in modulating protein activity (183). The highly dynamic nature of the ECM plays a crucial role in cancer progression and is the first barrier to developing metastasis. ECM remodeling is hijacked by tumor cells and invading stromal cells, resulting in dysregulated remodeling and dynamics (184, 185). This alters the composition and organization of the ECM and eventually leads to changes in its essential properties (23, 25). However, the exact interactions and the role of BM components such as perlecan in mediating the abnormalities remain unstudied.

The breaching mechanism by which tumor cells invade the BM has not been clearly determined but has been proposed to involve a number of ECM-distinct and most likely complementary mechanisms: proteolytic degradation of the ECM in parallel with abnormal ECM synthesis (186). Degradation of ECM is mediated by multiple proteases including MMPs, ADAMs, and ADAM-TS (short for a disintegrin and metalloproteinase, and a disintegrin and metalloproteinase with thrombospondin motifs), in addition to heparanase, liberating pro-angiogenic factors that in turn activate angiogenesis and promote the proliferation of tumor cells (185, 187). Stromal cells, including CAFs, along with infiltrating immune cells and tumor cells, results in a sustained presence of these proteinases. This situation overall leads to the progressive destruction of normal ECM and establishment of the cancer-associated ECM. Remarkably, it is the same set of proteins, in different structural configurations and likely altered interactions with each other and the surrounding environment, that results in the abnormal ECM. Certain regions within the ECM have been identified to be important for tumor cell proliferation and survival but can be partially hidden or “cryptic;” only becoming unmasked upon enzymatic digestion (142). At present, no cryptic epitopes have been identified for perlecan but undoubtedly the fine structural sequences of the HS chains may be accountable.

#### Angiogenesis

Angiogenesis is a key requirement for cancer growth and progression (188); this multi-step process is dependent on ECM remodeling and endothelial cell activation for the coordinated differentiation into functional vessels. HSPGs have long been acknowledged to control angiogenesis via the sequestering and release of growth factors which regulate endothelial cells, smooth muscle cells, and fibroblasts (189). The role of perlecan in pro-angiogenetic and anti-angiogenic functions place it center stage. Both tumor cells and host stromal cells synthesize perlecan; confirmed by a series of early xenograft immunostaining and transcriptional studies (166, 168, 190). The secretion of perlecan by tumor cells was proposed by the authors to facilitate formation of blood vessels during tumor expansion through the binding and interaction between perlecan and angiogenic growth factors. The incorporation of tumor perlecan into host blood vessels is likely mobilized by proteases easing the recruitment and diffusion of angiogenic growth factors into the tumor stroma (89). Gradients of perlecan expression have been observed in tumor vessels with the most reactive areas located at or around the sprouting edges, suggesting that tumor-derived perlecan can favor or induce the neovascularization of tumors (166, 190). Alternatively, host cells are proposed to synthesize perlecan as a defensive mechanism, with HS acting as a “sink” for growth factors by limiting their diffusion (154). The HS chains may be key elements that direct the intermolecular interactions that occur between perlecan and other BM components. The diverse substructure of HS chains might influence not only the growth factor-binding ability of perlecan but mediate roles in adhesion that can affect cancer cell proliferation and migration (86).

Tumor cells can also upregulate the production of several angiogenic factors such as FGF and VEGF in order to support their altered growth patterns and metabolism (154). For example, tumor vessels formed as a result of VEGF upregulation are abnormal; these vessels are variably fenestrated and leaky, accompanied by a disorganized or loose BM (191) (Figure 1, Box 2). These conditions typically lead to high interstitial pressures, escalated tissue hypoxia and production of additional VEGF (192). Human prostate cancer cells, depleted of perlecan and grafted in mice, produced tumors of decreased size and vascularization, where the effects were correlated to reduced secretion of VEGF-A in the xenografts (193). The occurrence of hypoxia during the early stages of tumor growth has been shown to regulate a number of angiogenic growth factors and cytokines, including VEGF (194). The expression of regulatory enzymes responsible for HS chain synthesis is also subject to hypoxic influence with preferential synthesis of HS resulting in increased responsiveness of hypoxic endothelial cells to FGF-2 (195). The release of heparanase from tumor cells into the ECM promotes cleavage of HS fragments, which in turn liberates bound growth factors that act to further support tumor angiogenesis (196). Perlecan also plays a role in establishing cytokine gradients in the ECM which are utilized by cells to migrate through tissues, as in the case of angiogenesis (87, 197).

#### Invasion

Malignant tumors are characterized by their invasiveness into nearby tissues, followed by metastasis to distal locations away from the primary tumor site. In order for these processes to take place, a series of signaling mechanisms contribute to the breakdown of the surrounding ECM by activating or releasing various proteolytic enzymes. A key enzyme involved in HSPG processing is heparanase, which recognizes a HS sulfation motif to hydrolyze the glycosidic bond between glucuronic acid and glucosamine (198). Heparanase activity digests HSPGs, resulting in increased endothelial permeability that enables the passage of invading cells through established boundaries, and the release of sequestered growth factors and soluble HS fragments that support angiogenesis and tumor growth (196). It has also been proposed that reduced adhesion of tumor cells to the underlying ECM, as well as increased cell motility, is due to cleavage of cell surface HS by heparanase produced by the tumor cell itself (108). Notably, heparanase has also been recognized to participate in some non-enzymatic activities, separate from its involvement in ECM degradation and remodeling (199–201).

Upregulation of heparanase occurs in essentially all human tumors and is closely correlated with an invasive phenotype in experimental models and has been linked to worse outcomes in cancer patients (196, 202, 203). A few examples are presented. Lung metastatic melanoma cells overexpress heparanase isoform 1 (Hpa1) mRNA (up to 29-fold) compared to normal lung tissue (204). Hpa1 enzyme was identified around vascularized regions, as well as blood vessels near the invasion front in various representative models (204, 205). Heparanase over-expressing breast tumors are seven times larger and present significantly more vascularization (206). Friedmann et al. presented high levels of heparanase mRNA in lymph, liver, and lung tumor metastases with the highest amounts of both mRNA and enzyme detected in deeply invading colon carcinoma cells (207). Heparanase activity is upregulated in lung and brain cancers, with melanoma cells that are highly metastatic to the brain overexpressing Hpa1 (208, 209). Specimens from breast cancer patients showed that lymphocytes express heparanase and when serum collected from these patients was introduced to fresh lymphocytes, heparanase expression was stimulated in the normal lymphocytes (210). Furthermore, a non-metastatic cell type, transfected with the gene that encodes heparanase, acquired a metastatic phenotype (211). Hypoxia was found to augment heparanase activity and consequently invasion in ovarian cancer cell lines (212). Inversely, anti-sense targeting of heparanase weakens the invasive ability of carcinoma cells (213).

The importance of HSPG structure in tumor biology was demonstrated in a study where Liu et al. injected bacterial recombinant heparinase (Hep) I (which cleaves highly sulfated regions) and Hep III (which cleaves unsulfated regions) into melanoma challenged mice and found that the specificity of the enzymes dictated whether tumors regressed (Hep III) or advanced (Hep I) due to where the different enzymes cleaved HS (214). This finding demonstrated both the heterogeneity of HS and the fine control of biological function due to these different HS structures. They found that the resulting tumor cell GAG fragments were distinct following treatment with the different heparinase isoforms, with Hep III digestion causing up to 75% inhibition in tumor growth whereas fragments as a result of Hep I digestion significantly enhanced growth. Furthermore, the demonstrated effects were modulated by FGF-2 signaling, as Hep I-generated fragments promoted FGF-2 activity, whereas Hep III-generated fragments inhibited signaling, with additional implication of MAP kinase and FAK pathways. It should be noted that there is a difference in the mechanism by which mammalian-derived heparanase and bacterial-derived heparinase cleave HS; heparanase is a hydrolase, as opposed to heparinase which is an eliminase (215). In some instances, the overexpression of heparanase is linked to other enzyme activities. In addition to heparanase overexpression, melanoma cells were reported to exhibit 3-O-sulfotransferase gene hypermethylation and subsequent gene silencing (216). A study by Ma and Geng, showed that the cell adhesion molecule P-selectin, present on endothelial cells and activated platelets, was still capable of binding to a HS-like molecule displayed on melanoma cells despite the absence of its recognition motif (217). Interplay between a series of enzymes including 3-, 6-O-sulfotransferase and HSulf enzymes may transform HS to confer P-selectin binding ability and hence promote the migration of cells to secondary sites (81). Additionally, heparanase mediates upregulation of MMP-9, expressed from tumor cells, to indirectly stimulate invasion (218). In addition to the biological effects modulated by the HS chains of perlecan, perlecan-rich borders can resist cell invasion and serve as tissue boundaries (107). These borders include the glandular BM (219), the reactive stromal compartment (173), the vasculature (220), and bone marrow reticular matrix (221). Perlecan and MMP-7 co-localize at tissue boundaries when surveyed in prostate cancer sections, with MMP-7 proposed to act as a molecular switch by altering cancer cell behavior to favor cell dispersion and invasiveness (222, 223).

While increased expression of perlecan is shown in a number of tumor types (Table 1), its levels are also undetectable in other instances. Several early studies reported strong mRNA levels of perlecan with the overexpressed perlecan protein deposited in the ECM and in tumor cells at the invading front (164, 171, 172, 224). These studies were supported by observations of inhibited tumor growth and angiogenesis (193, 225) or reduced cell proliferation and invasiveness (226) when perlecan was downregulated by anti-sense targeting. This is contrary to the findings reported by Mathiak et al. where anti-sense targeting of perlecan resulted in stimulation of tumor cell growth *in vitro* and *in vivo* accompanied with increased invasiveness in the ECM (227). It has been suggested that the lack of perlecan in these cases could perhaps be related to the tissue microenvironment preferentially favoring the diffusion of growth factors, which encourages tumor growth and metastasis (142, 154). Alternatively, Nerlich et al. reported high levels of perlecan mRNA in both tumor and stromal cells but then very low levels of perlecan protein present in tumor-associated BM (168, 169). Similarly, differences were observed between perlecan mRNA and secreted protein measured from stably transfected anti-sense perlecan targeting subclones, with reduction of >50% compared to the untransfected parental cell line (193). A recent study exploring the localization of perlecan in squamous cell carcinoma (SCC) reveals that perlecan and its binding growth factors namely VEGF [binds to HS chains (85)], Sonic Hedgehog (SHH) [HS and protein core (228)], and FGF-7 [protein core (147)] co-localize within the epithelial layer before invasion (176). Once the carcinoma cells started to invade, perlecan and FGF-7 were identified in the stromal space while VEGF and SHH remained at the epithelial layer. This correlates with other studies that suggested biosynthesis of perlecan was switched over from carcinoma cells to stromal cells (174, 190, 229, 230). The discrepancy between significantly enhanced mRNA synthesis and loss in protein deposition may also point to the activity of proteolytic enzymes or a post-translational block of protein synthesis or both (154).

Overexpression of perlecan in prostate cancer stroma has been linked to TNFα-mediated transcriptional induction (173). This suggests that perlecan transcription could be a part of cytokine-mediated innate immune response to cancer invasion. Perlecan has also been implicated in regulating prostate cancer progression via the SHH pathway (231). Franses and colleagues explored the role of endothelial cells in regulating cancer cell behavior, where perlecan silencing eliminated the ability of endothelial cells to suppress cancer invasiveness in both *in vitro* and *in vivo* models of breast and lung cancer (75). These findings indirectly contrast with the early work (discussed above) showing that perlecan depletion (albeit in cancer cells) slows tumor growth and reduces metastasis (193, 225, 226). The fact that perlecan acts in a cell context-specific manner could be a consideration for the contradicting data (142). It is important to note that perlecan derived from different cellular sources carries different HS structures and as such different growth factor binding and functional capabilities (94, 157). For example, Lord and colleagues have shown that the GAG chains differ between perlecan enriched from human coronary artery smooth muscle or endothelial cells and this influences their roles in mediating cell adhesion and proliferation, as well as FGF binding and signaling (157). Therefore, it can be summarized that tumor subtype, stage, degree of tumor differentiation, and/or various histological location and identifying reagent (i.e., primer region of interest or antibody epitope) may result in the different distribution of perlecan across the reported studies.

## Therapeutic Targeting of Heparan Sulfate Proteoglycans and Their Function in Cancer Metastasis

Therapies that target HSPGs in cancer metastasis cover a range of modalities, highlighted in Table 2. Most therapies that target metastasis and the role of HS revolve around the inhibition of heparanase. The inhibition of heparanase eliminates the cleavage of HS chains and the release of bioactive molecules such as, FGFs, and VEGF, to disrupt the downstream events that are associated not only with the progression of cancer but also with cancer metastasis. Given the prevalence of cancer and the role of HSPGs in multiple events there is an extensive amount of literature, including a number of recent reviews (203, 247, 248), that detail the mechanisms of action of the range of therapeutics that are being developed. The following section will review the most recent advances in the field.

**Table 2:** Summary of therapeutics that target heparan sulfate proteoglycans.

**Therapeutic**	**Results or observations (specific compound reported in brackets)**	**References**
HS mimetic/ heparanase inhibitor	In a Phase I clinical trial demonstrated safety though anti-myeloma efficacy was minimal (Roneparstat)	(232)
	Demonstrated safety in a Phase I clinical trial for melanoma [Muparfostat (PI-88)]	(233)
	Acceptable safety and encouraging signals of activity in patients with metastatic pancreatic cancer in Phase I clinical trial [Neuparanib (N-402)]	(234)
	Anti-metastatic effects in murine models of melanoma and lung cancer	(235)
	Inhibition of primary tumor growth and reduced metastasis in murine breast cancer model	(236)
	Acceptable safety and encouraging signals of activity in patients with metastatic pancreatic cancer in Phase I clinical trial	(234)
	Inhibition of metastasis from primary tumor in a lung cancer patient derived xenograft model	(237)
	Reduced MMP1 expression and increased TIMP3 expression in pancreatic cancer patients	(238)
LMWH	Reduced primary tumor and pulmonary metastasis in a murine melanoma model. LMWH was incorporated into a hydrogel system	(239)
Heparanase inhibitor	Benzoxazole derivatives demonstrated anti-metastatic potential via reduced expression levels of FGF-1, FGF-2, VEGF, and MMP-3 in a fibrosarcoma derived cell line	(240)
Sulfatase inhibitor	Inhibition of TGFβ1/SMAD and Hedgehog/GL1 pathways in hepatocellular carcinoma cell lines	(241)
	Reduced tumor size in mice implanted with xenograft pediatric glioblastomas	(242)
Immunotherapy	GPC-2 targeting antibody-drug conjugate reduced proliferation of GPC-2 expressing cells derived from neuroblastomas	(243)
	Monoclonal antibody that binds to GPC-3 demonstrated safety in a Phase I clinical trial for hepatocellular carcinoma	(244)
	GPC-3 CAR-T cells eliminated GPC-3 positive tumors in murine model of hepatocellular carcinoma.	(245, 246)

The first reports of heparanase inhibitors in an anti-cancer or anti-metastatic activity, stemmed from the use of heparin and low molecular weight heparins (LMWHs) (249). As heparin has a similar structure to HS, though a higher sulfated version, it competes with endogenous HS for both heparanase binding and substrate activity. However, the risk to patients regarding bleeding due to anticoagulant activity of heparin has limited their use as therapeutics for cancer and cancer metastasis, particularly as a long term therapeutic. Given the potential of both heparin and LMWHs, much effort has been directed toward either modifying or mimicking the structure heparin/LMWHs to remove the anticoagulant activity whilst retaining the ability to inhibit heparanase. The success of HS mimetics is clear through the number of these materials that have made it through to clinical trials. Modification of heparin through desulfation and glycol splitting has seen the development of roneparstat (250) and its investigation in a Phase I trial as a therapeutic for myeloma (232). In addition to roneparastat, HS mimetics muparfostat (PI-88) (233), neuparanib (N-402) (234), piixatimod (PG545) (251), have been, or are currently in clinical trials for use as a therapy targeting metastasis of melanoma or pancreatic cancer. More recent reports have detailed the use of these HS mimetics not only in the development of therapeutics, but the development of more representative models for testing anti-cancer/anti-metastatic therapeutics including patient-derived xenografts (237) and organoid models (238). Neuparanib has been shown to reduce tumor cell proliferation and invasion in an organoid model, and plasma levels of patients within a clinical trial cohort reported increased levels of tissue inhibitor of MMP-3 (238). The attempt at mimicking the structure of HS has seen the development of glycopolymers with well-defined sulfation patterns and the ability to optimize disaccharide length for peak heparanase inhibition (252), which reduced metastasis of breast cancer in a rodent model. The ability to design and synthesize HS mimicking structures that eliminate anti-coagulation activity and target heparanase has more recently been facilitated with use of computational modeling to predict the anti-cancer/anti-metastatic potential (253–255).

In addition to the issues associated with anticoagulant activity, heparin also has a short half-life which can mean when administered intravenously that high dosages are required for a therapeutic effect or that there is the need for multiple injections. More recent reports have demonstrated the therapeutic use of heparin via incorporation or tethering to a substrate for targeted delivery. Reduction of metastasis in a lung cancer model was achieved with incorporation of heparin into a hydrogel system for local administration of the therapeutic (256). Tethering heparin to oligonucleotides via a cleavable linker that is pH sensitive (239), has also been demonstrated as a method of targeted delivery and the reduction of pulmonary metastasis in a melanoma model. Furthermore, delivery of LMWH, through tethering to micelles, reduced pulmonary metastasis in a breast cancer model, which was further reduced by using a delivery system that facilitated targeted co-delivery of the LMWH with the chemotherapy agent doxorubicin (257).

Despite their anti-metastatic properties, HS mimetics and polysaccharide derivatives have limitations due to their relatively high molecular weights, and rather heterogenous structures. More recently, there has been the exploration of small molecular inhibitors of heparanase, that overcome these limitations, for example benzimidazole and benzoxazole derivatives (258–260). Benzimidazole and benzoxazole derivates have been long studied in medicinal chemistry (261). Most recent advances in these derivatives include the synthesis of symmetrical analogs that demonstrated superior anti-heparanase activity as compared to non-symmetrical analogs (240), with the ability to not only inhibit heparanase, but also bind and sequester HS interacting growth factors and chemokines that modulate angiogenesis.

In addition to heparanase, sulfatases can modify HS via the removal of 6-*O*-sulfate groups and as such have been investigated as a targeting molecule. The compound designated OK-007, 2,4-disulfophenyl-*N*-tert-butylnitrone, inhibits the enzymatic activity of Sulf2. This compound was initially explored as a treatment for acute ischemic stroke (262), though has since been investigated as a potential therapeutic for HCC (241) and glioblastoma (242). Coutinho de Souza et al. (242) demonstrated the ability for OKN-007 to reduce cell proliferation and the expression of the receptor for platelet derived growth factor, and the authors speculated potential anti-angiogenic properties of OKN-007.

More recently, monoclonal antibody therapy, a form of immunotherapy, has been explored as a route to target HSPGs. Though, these therapies have been mainly focused toward targeting primary rather than secondary tumors. Monoclonal antibodies targeting GPC-2 have been developed as a therapeutic for neuroblastoma (243), and antibodies targeting GPC-3 have progressed to phase I trials in HCC (244). More recently GPC-3 in HCC has been used as a target in chimeric antigen receptor, or CAR T-cell therapy (245, 246, 263), with the therapy demonstrating the ability to reduced HCC tumors in a xenograft model (245).

## Conclusions

The role of HSPGs in cancer metastasis is through the interaction of the HS chains or PG protein core with key biological molecules associated with metastatic events. The non-templated heterogeneous structure of HS modulates these specific interactions between mediators, influencing events in the metastatic cascade. Furthermore, the increase in heparanase expression in multiple cancer types results in the cleavage of HS chains and release of mediators involved in these events. HSPGs, including perlecan, have antithetic roles in cancer and metastasis through the interaction with biological molecules. The subtle differences in HSPG structure, particularly that of HS, results in a family of molecules that behave as both pro- or anti-metastatic factors. Thus, due to the structure specific interactions between HS and mediators of metastatic events, future therapeutics that target HSPGs and their cleaving enzymes need to target specific HS or heparanase binding structures, and ideally have targeted delivery, to ensure both efficacy and reduced off-target effects to truly improve patient outcomes.

## Author Contributions

All authors contributed to the reviewing the literature and writing of this manuscript.

### Conflict of Interest

The authors declare that the research was conducted in the absence of any commercial or financial relationships that could be construed as a potential conflict of interest.

## References

[1] Steeg PS. Tumor metastasis: mechanistic insights and clinical challenges. Nat Med. (2006) 12:895–904. 10.1038/nm1469 16892035

[2] Bray F Ferlay J Soerjomataram I Siegel RL Torre LA Jemal A. Global cancer statistics 2018: GLOBOCAN estimates of incidence and mortality worldwide for 36 cancers in 185 countries. CA Cancer J Clin. (2018) 68:394–424. 10.3322/caac.21492 30207593

[3] Vogelstein B Kinzler KW. The multistep nature of cancer. Trends Genet. (1993) 9:138–41. 10.1016/0168-9525(93)90209-Z 8516849

[4] Vogelstein B Kinzler KW. Cancer genes and the pathways they control. Nat Med. (2004) 10:789–99. 10.1038/nm1087 15286780

[5] Bernards R Weinberg RA Metastasis genes: a progression puzzle. Nature. (2002) 418:823 10.1038/418823a 12192390

[6] Priestley P Baber J Lolkema MP Steeghs N de Bruijn E Shale C. Pan-cancer whole-genome analyses of metastatic solid tumours. Nature. (2019) 575:210–6. 10.1038/s41586-019-1689-y 31645765 PMC6872491

[7] Chitty JL Filipe EC Lucas MC Herrmann D Cox TR Timpson P. Recent advances in understanding the complexities of metastasis. F1000Research. (2018) 7:F1000. 10.12688/f1000research.15064.2 30135716 PMC6073095

[8] Hanahan D Robert Weinberg A. Hallmarks of cancer: the next generation. Cell. (2011) 144:646–74. 10.1016/j.cell.2011.02.013 21376230

[9] Quail DF Joyce JA. Microenvironmental regulation of tumor progression and metastasis. Nat Med. (2013) 19:1423. 10.1038/nm.3394 24202395 PMC3954707

[10] Barker HE Cox TR Erler JT. The rationale for targeting the LOX family in cancer. Nat Rev Cancer. (2012) 12:540–52. 10.1038/nrc3319 22810810

[11] Filipe EC Chitty JL Cox TR. Charting the unexplored extracellular matrix in cancer. Int J Exp Pathol. (2018) 99:58–76. 10.1111/iep.12269 29671911 PMC6031881

[12] Cox TR Erler JT. Remodeling and homeostasis of the extracellular matrix: implications for fibrotic diseases and cancer. Dis Models Mech. (2011) 4:165–78. 10.1242/dmm.004077 21324931 PMC3046088

[13] Mouw JK Ou G Weaver VM. Extracellular matrix assembly: a multiscale deconstruction. Nat Rev Mol Cell Biol. (2014) 15:771–85. 10.1038/nrm3902 25370693 PMC4682873

[14] Pereira BA Vennin C Papanicolaou M Chambers CR Herrmann D Morton JP. CAF Subpopulations: a new reservoir of stromal targets in pancreatic cancer. Trends Cancer. (2019) 5:724–41. 10.1016/j.trecan.2019.09.010 31735290

[15] Boyle ST Kular J Nobis M Ruszkiewicz A Timpson P Samuel MS. Acute compressive stress activates RHO/ROCK-mediated cellular processes. Small GTPases. (2018) 10.1080/21541248.2017.1413496. [Epub ahead of print]. 29455593 PMC7549670

[16] El-Haibi CP Bell GW Zhang J Collmann AY Wood D Scherber CM. Critical role for lysyl oxidase in mesenchymal stem cell-driven breast cancer malignancy. Proc Natl Acad Sci USA. (2012) 109:17460–5. 10.1073/pnas.1206653109 23033492 PMC3491529

[17] Heldin P Basu K Olofsson B Porsch H Kozlova I Kahata K. Deregulation of hyaluronan synthesis, degradation and binding promotes breast cancer. J Biochem. (2013) 154:395–408. 10.1093/jb/mvt085 24092768

[18] Venning FA Wullkopf L Erler JT. Targeting ECM disrupts cancer progression. Front Oncol. (2015) 5:224. 10.3389/fonc.2015.00224 26539408 PMC4611145

[19] Nagaharu K Zhang X Yoshida T Katoh D Hanamura N Kozuka Y. Tenascin C induces epithelial-mesenchymal transition-like change accompanied by SRC activation and focal adhesion kinase phosphorylation in human breast cancer cells. Am J Pathol. (2011) 178:754–63. 10.1016/j.ajpath.2010.10.015 21281808 PMC3069868

[20] Yoshida T E-Matsumoto I Hanamura N Kalembeyi I Katsuta K Ishihara A. Co-expression of tenascin and fibronectin in epithelial and stromal cells of benign lesions and ductal carcinomas in the human breast. J Pathol. (1997) 182:421–8. 10.1002/(SICI)1096-9896(199708)182:4<421::AID-PATH886>3.3.CO;2-L 9306963

[21] Mennerich D Vogel A Klaman I Dahl E Lichtner RB Rosenthal A. Shift of syndecan-1 expression from epithelial to stromal cells during progression of solid tumours. Eur J Cancer. (2004) 40:1373–82. 10.1016/j.ejca.2004.01.038 15177497

[22] Shen M Jiang YZ Wei Y Ell B Sheng X Esposito M. Tinagl1 suppresses triple-negative breast cancer progression and metastasis by simultaneously inhibiting integrin/FAK and EGFR signaling. Cancer Cell. (2019) 35:64–80.e7. 10.1016/j.ccell.2018.11.016 30612941

[23] Pickup MW Mouw JK Weaver VM. The extracellular matrix modulates the hallmarks of cancer. EMBO Rep. (2014) 15:1243–53. 10.15252/embr.201439246 25381661 PMC4264927

[24] Kai F Laklai H Weaver VM. Force matters: biomechanical regulation of cell invasion and migration in disease. Trends Cell Biol. (2016) 26:486–97. 10.1016/j.tcb.2016.03.007 27056543 PMC4970516

[25] Lu P Weaver VM Werb Z. The extracellular matrix: a dynamic niche in cancer progression. J Cell Biol. (2012) 196:395–406. 10.1083/jcb.201102147 22351925 PMC3283993

[26] Weaver VM Petersen OW Wang F Larabell CA Briand P Damsky C. Reversion of the malignant phenotype of human breast cells in three-dimensional culture and *in vivo* by integrin blocking antibodies. J Cell Biol. (1997) 137:231. 10.1083/jcb.137.1.231 9105051 PMC2139858

[27] Conklin MW Eickhoff JC Riching KM Pehlke CA Eliceiri KW Provenzano PP. Aligned collagen is a prognostic signature for survival in human breast carcinoma. Am J Pathol. (2011) 178:1221–32. 10.1016/j.ajpath.2010.11.076 21356373 PMC3070581

[28] Provenzano PP Eliceiri KW Campbell JM Inman DR White JG Keely PJ. Collagen reorganization at the tumor-stromal interface facilitates local invasion. BMC Med. (2006) 4:38. 10.1186/1741-7015-4-38 17190588 PMC1781458

[29] Martinez B Yang Y Harker DMR Farrar C Mukundan H Nath P. YAP/TAZ Related biomechano signal transduction and cancer metastasis. Front Cell Dev Biol. (2019) 7:199. 10.3389/fcell.2019.00199 31637239 PMC6788381

[30] Northcott JM Dean IS Mouw JK Weaver VM. Feeling stress: the mechanics of cancer progression and aggression. Front Cell Dev Biol. (2018) 6:17. 10.3389/fcell.2018.00017 29541636 PMC5835517

[31] Shields JD Fleury ME Yong C Tomei AA Randolph GJ Swartz MA. Autologous chemotaxis as a mechanism of tumor cell homing to lymphatics via interstitial flow and autocrine CCR7 signaling. Cancer Cell. (2007) 11:526–38. 10.1016/j.ccr.2007.04.020 17560334

[32] Friedl P Wolf K. Proteolytic interstitial cell migration: a five-step process. Cancer Metastasis Rev. (2009) 28:129–35. 10.1007/s10555-008-9174-3 19153672

[33] van Helvert S Storm C Friedl P. Mechanoreciprocity in cell migration. Nat Cell Biol. (2017) 20:8–20. 10.1038/s41556-017-0012-0 29269951 PMC5943039

[34] Yu Q Stamenkovic I. Cell surface-localized matrix metalloproteinase-9 proteolytically activates TGF-β and promotes tumor invasion and angiogenesis. Genes Dev. (2000) 14:163–76. 10.1101/gad.14.2.163 10652271 PMC316345

[35] Hosseini H Obradović MMS Hoffmann M Harper KL Sosa MS Werner-Klein M. Early dissemination seeds metastasis in breast cancer. Nature. (2016) 540:552–8. 10.1038/nature20785 27974799 PMC5390864

[36] Harper KL Sosa MS Entenberg D Hosseini H Cheung JF Nobre R. Mechanism of early dissemination and metastasis in Her2(+) mammary cancer. Nature. (2016) 540:588–92. 10.1158/1538-7445.AM2017-3051 27974798 PMC5471138

[37] Chang TT Thakar D Weaver VM. Force-dependent breaching of the basement membrane. Matrix Biol. (2017) 57–58:178–89. 10.1016/j.matbio.2016.12.005 28025167 PMC5328923

[38] Kelley LC Lohmer LL Hagedorn EJ Sherwood DR. Traversing the basement membrane *in vivo*: a diversity of strategies. J Cell Biol. (2014) 204:291–302. 10.1083/jcb.201311112 24493586 PMC3912525

[39] Sabeh F Shimizu-Hirota R Weiss SJ. Protease-dependent versus -independent cancer cell invasion programs: three-dimensional amoeboid movement revisited. J Cell Biol. (2009) 185:11–9. 10.1083/jcb.200807195 19332889 PMC2700505

[40] Ecker BL Kaur A Douglass SM Webster MR Almeida FV Marino GE. Age-related changes in HAPLN1 increase lymphatic permeability and affect routes of melanoma metastasis. Cancer Discov. (2019) 9:82–95. 10.1158/2159-8290.CD-18-0168 30279172 PMC6328344

[41] Kaur A Ecker BL Douglass SM Kugel CH Webster MR Almeida FV. Remodeling of the collagen matrix in aging skin promotes melanoma metastasis and affects immune cell motility. Cancer Discov. (2019) 9:64–81. 10.1158/2159-8290.CD-18-0193 30279173 PMC6328333

[42] Singleton PA Dudek SM Ma SF Garcia JG. Transactivation of sphingosine 1-phosphate receptors is essential for vascular barrier regulation. Novel role for hyaluronan and CD44 receptor family. J Biol Chem. (2006) 281:34381–93. 10.1074/jbc.M603680200 16963454

[43] Singleton PA Mirzapoiazova T Guo Y Sammani S Mambetsariev N Lennon FE. High-molecular-weight hyaluronan is a novel inhibitor of pulmonary vascular leakiness. Am J Physiol Lung Cell Mol Physiol. (2010) 299:L639–51. 10.1152/ajplung.00405.2009 20709728 PMC2980391

[44] Mambetsariev N Mirzapoiazova T Mambetsariev B Sammani S Lennon FE Garcia JG. Hyaluronic Acid binding protein 2 is a novel regulator of vascular integrity. Arterioscler Thromb Vasc Biol. (2010) 30:483–90. 10.1161/ATVBAHA.109.200451 20042707 PMC2825278

[45] Koliopanos A Friess H Kleeff J Shi X Liao Q Pecker I. Heparanase expression in primary and metastatic pancreatic cancer. Cancer Res. (2001) 61:4655. Available online at: https://cancerres.aacrjournals.org/content/61/12/4655 11406531

[46] Kurokawa H Katsube KI Podyma KA Ikuta M Iseki H Nakajima M. Heparanase and tumor invasion patterns in human oral squamous cell carcinoma xenografts. Cancer Sci. (2003) 94:277–85. 10.1111/j.1349-7006.2003.tb01433.x 12824922 PMC11160257

[47] Eccles SA. Heparanase: breaking down barriers in tumors. Nat Med. (1999) 5:735–6. 10.1038/10455 10395313

[48] Douma S Van Laar T Zevenhoven J Meuwissen R Van Garderen E Peeper DS Suppression of anoikis and induction of metastasis by the neurotrophic receptor Trk. Nature B. (2004) 430:1034–9. 10.1038/nature02765 15329723

[49] Yu M Ting DT Stott SL Wittner BS Ozsolak F Paul S. RNA sequencing of pancreatic circulating tumour cells implicates WNT signalling in metastasis. Nature. (2012) 487:510–3. 10.1038/nature11217 22763454 PMC3408856

[50] Duda DG Duyverman MJ Kohno M Snuderl ME Steller JA Fukumura D. Malignant cells facilitate lung metastasis by bringing their own soil. Proc Natl Acad Sci USA. (2010) 107:21677–82. 10.1073/pnas.1016234107 21098274 PMC3003109

[51] Labelle M Begum S Richard Hynes O. Direct signaling between platelets and cancer cells induces an epithelial-mesenchymal-like transition and promotes metastasis. Cancer Cell. (2011) 20:576–90. 10.1016/j.ccr.2011.09.009 22094253 PMC3487108

[52] Palumbo JS. Platelets and fibrin(ogen) increase metastatic potential by impeding natural killer cell-mediated elimination of tumor cells. Blood. (2005) 105:178–85. 10.1182/blood-2004-06-2272 15367435

[53] Palumbo JS Talmage KE Massari JV La Jeunesse CM Flick MJ Kombrinck KW. Tumor cell-associated tissue factor and circulating hemostatic factors cooperate to increase metastatic potential through natural killer cell-dependent and-independent mechanisms. Blood. (2007) 110:133–41. 10.1182/blood-2007-01-065995 17371949 PMC1896107

[54] Peinado H Zhang H Matei IR Costa-Silva B Hoshino A Rodrigues G. Pre-metastatic niches: organ-specific homes for metastases. Nat Rev Cancer. (2017) 17:302–17. 10.1038/nrc.2017.6 28303905

[55] Chen W Hoffmann AD Liu H Liu X. Organotropism: new insights into molecular mechanisms of breast cancer metastasis. NPJ Precis Oncol. (2018) 2:4. 10.1038/s41698-018-0047-0 29872722 PMC5871901

[56] Huang Y Song N Ding Y Yuan S Li X Cai H. Pulmonary vascular destabilization in the premetastatic phase facilitates lung metastasis. Cancer Res. (2009) 69:7529–37. 10.1158/0008-5472.CAN-08-4382 19773447

[57] Yan HH Pickup M Pang Y Gorska AE Li Z Chytil A. Gr-1+CD11b+ Myeloid cells tip the balance of immune protection to tumor promotion in the premetastatic lung. Cancer Res. (2010) 70:6139–49. 10.1158/0008-5472.CAN-10-0706 20631080 PMC4675145

[58] Kaplan RN Rafii S Lyden D. Preparing the “soil”: the premetastatic niche. Cancer Res. (2006) 66:11089–93. 10.1158/0008-5472.CAN-06-2407 17145848 PMC2952469

[59] Murgai M Ju W Eason M Kline J Beury DW Kaczanowska S. KLF4-dependent perivascular cell plasticity mediates pre-metastatic niche formation and metastasis. Nat Med. (2017) 23:1176–90. 10.1038/nm.4400 28920957 PMC5724390

[60] Kaplan RN Riba RD Zacharoulis S Bramley AH Vincent L Costa C. VEGFR1-positive haematopoietic bone marrow progenitors initiate the pre-metastatic niche. Nature. (2005) 438:820–7. 10.1038/nature04186 16341007 PMC2945882

[61] Chiarugi P Giannoni E. Anoikis: a necessary death program for anchorage-dependent cells. Biochem Pharmacol. (2008) 76:1352–64. 10.1016/j.bcp.2008.07.023 18708031

[62] Shibue T Weinberg RA. Integrin beta1-focal adhesion kinase signaling directs the proliferation of metastatic cancer cells disseminated in the lungs. Proc Natl Acad Sci USA. (2009) 106:10290–5. 10.1073/pnas.0904227106 19502425 PMC2700942

[63] Cox TR Bird D Baker AM Barker HE Ho MW Lang G. LOX-mediated collagen crosslinking is responsible for fibrosis-enhanced metastasis. Cancer Res. (2013) 73:1721–32. 10.1158/0008-5472.CAN-12-2233 23345161 PMC3672851

[64] Rath N Morton JP Julian L Helbig L Kadir S McGhee EJ. ROCK signaling promotes collagen remodeling to facilitate invasive pancreatic ductal adenocarcinoma tumor cell growth. EMBO Mol Med. (2017) 9:198–218. 10.15252/emmm.201606743 28031255 PMC5286371

[65] Vennin C Chin VT Warren SC Lucas MC Herrmann D Magenau A. Transient tissue priming via ROCK inhibition uncouples pancreatic cancer progression, sensitivity to chemotherapy, and metastasis. Sci Transl Med. (2017) 9:eaai8504. 10.1126/scitranslmed.aai8504 28381539 PMC5777504

[66] Oskarsson T Acharyya S Zhang XHF Vanharanta S Tavazoie SF Morris PG. Breast cancer cells produce tenascin C as a metastatic niche component to colonize the lungs. Nat Med. (2011) 17:867–74. 10.1038/nm.2379 21706029 PMC4020577

[67] Malanchi I Santamaria-Martínez A Susanto E Peng H Lehr HA Delaloye JF. Interactions between cancer stem cells and their niche govern metastatic colonization. Nature. (2011) 481:85–9. 10.1038/nature10694 22158103

[68] Gao H Chakraborty G Ai Lee-Lim P Mo Q Decker M Vonica A. The BMP inhibitor coco reactivates breast cancer cells at lung metastatic sites. Cell. (2012) 150:764–79. 10.1016/j.cell.2012.06.035 22901808 PMC3711709

[69] Bui AT Laurent F Havard M Dautry F Tchénio T. SMAD signaling and redox imbalance cooperate to induce prostate cancer cell dormancy. Cell Cycle. (2015) 14:1218–31. 10.1080/15384101.2015.1014145 25706341 PMC4615032

[70] Kobayashi A Okuda H Xing F Pandey PR Watabe M Hirota S. Bone morphogenetic protein 7 in dormancy and metastasis of prostate cancer stem-like cells in bone. J Exp Med. (2011) 208:2641–55. 10.1084/jem.20110840 22124112 PMC3244043

[71] Sharma S Xing F Liu Y Wu K Said N Pochampally R. Secreted protein acidic and rich in cysteine (SPARC) mediates metastatic dormancy of prostate cancer in bone. J Biol Chem. (2016) 291:19351–63. 10.1074/jbc.M116.737379 27422817 PMC5016675

[72] Ghajar CM Peinado H Mori H Matei IR Evason KJ Brazier H. The perivascular niche regulates breast tumour dormancy. Nat Cell Biol. (2013) 15:807–17. 10.1038/ncb2767 23728425 PMC3826912

[73] Kobayashi H Butler JM O'Donnell R Kobayashi M Ding BS Bonner B. Angiocrine factors from Akt-activated endothelial cells balance self-renewal and differentiation of haematopoietic stem cells. Nat Cell Biol. (2010) 12:1046–56. 10.1038/ncb2108 20972423 PMC2972406

[74] Franses JW Drosu NC Gibson WJ Chitalia VC Edelman ER. Dysfunctional endothelial cells directly stimulate cancer inflammation and metastasis. Int J Cancer. (2013) 133:1334–44. 10.1002/ijc.28146 23463345 PMC3707950

[75] Franses JW Baker AB Chitalia VC Edelman ER. Stromal endothelial cells directly influence cancer progression. Sci Transl Med. (2011) 3:66ra5. 10.1126/scitranslmed.3001542 21248315 PMC3076139

[76] Vennin C Melenec P Rouet R Nobis M Cazet A Murphy K CAF hierarchy governed by tumour cell p53-status creates pro-invasive and chemo-modulatory pancreatic stroma via perlecan. Nat Commun. (2019) 10:3637 10.1038/s41467-019-10968-6 31406163 PMC6691013

[77] Sasisekharan R Shriver Z Venkataraman G Narayanasami U. Roles of heparan-sulphate glycosaminoglycans in cancer. Nat Rev Cancer. (2002) 2:521–8. 10.1038/nrc842 12094238

[78] Afratis N Gialeli C Nikitovic D Tsegenidis T Karousou E Theocharis AD. Glycosaminoglycans: key players in cancer cell biology and treatment. FEBS J. (2012) 279:1177–97. 10.1111/j.1742-4658.2012.08529.x 22333131

[79] Fjeldstad K Kolset SO. Decreasing the metastatic potential in cancers–targeting the heparan sulfate proteoglycans. Curr Drug Targets. (2005) 6:665–82. 10.2174/1389450054863662 16178800

[80] Knelson EH Nee JC Blobe GC. Heparan sulfate signaling in cancer. Trends Biochem Sci. (2014) 39:277–88. 10.1016/j.tibs.2014.03.001 24755488 PMC4065786

[81] Raman K Kuberan B. Chemical tumor biology of heparan sulfate proteoglycans. Curr Chem Biol. (2010) 4:20–31. 10.2174/187231310790226206 20596243 PMC2892923

[82] Iozzo RV Sanderson RD. Proteoglycans in cancer biology, tumour microenvironment and angiogenesis. J Cell Mol Med. (2011) 15:1013–31. 10.1111/j.1582-4934.2010.01236.x 21155971 PMC3633488

[83] Gama CI Tully SE Sotogaku N Clark PM Rawat M Vaidehi N. Sulfation patterns of glycosaminoglycans encode molecular recognition and activity. Nat Chem Biol. (2006) 2:467–73. 10.1038/nchembio810 16878128

[84] Lindahl U Li JP. Interactions between heparan sulfate and proteins-design and functional implications. Int Rev Cell Mol Biol. (2009) 276:105–59. 10.1016/S1937-6448(09)76003-4 19584012

[85] Iozzo RV. Basement membrane proteoglycans: from cellar to ceiling. Nat Rev Mol Cell Biol. (2005) 6:646–56. 10.1038/nrm1702 16064139

[86] Whitelock JM Iozzo RV. Heparan sulfate: a complex polymer charged with biological activity. Chem Rev. (2005) 105:2745–64. 10.1021/cr010213m 16011323

[87] Sarrazin S Lamanna WC Esko JD. Heparan sulfate proteoglycans. Cold Spring Harb Perspect Biol. (2011) 3:a004952. 10.1101/cshperspect.a004952 21690215 PMC3119907

[88] Theocharis AD Skandalis SS Gialeli C Karamanos NK. Extracellular matrix structure. Adv Drug Deliv Rev. (2016) 97:4–27. 10.1016/j.addr.2015.11.001 26562801

[89] Whitelock JM Murdoch AD Iozzo RV Underwood PA. The degradation of human endothelial cell-derived perlecan and release of bound basic fibroblast growth factor by stromelysin, collagenase, plasmin, and heparanases. J Biol Chem. (1996) 271:10079–86. 10.1074/jbc.271.17.10079 8626565

[90] Bashkin P Razin E Eldor A Vlodavsky I. Degranulating mast cells secrete an endoglycosidase that degrades heparan sulfate in subendothelial extracellular matrix. Blood. (1990) 75:2204–12. 10.1182/blood.V75.11.2204.2204 1693299

[91] Wang B Jia J Zhang X Zcharia E Vlodavsky I Pejler G. Heparanase affects secretory granule homeostasis of murine mast cells through degrading heparin. J Allergy Clin Immunol. (2011) 128:1310–17.e8. 10.1016/j.jaci.2011.04.011 21575986 PMC3160500

[92] Bishop JR Schuksz M Esko JD. Heparan sulphate proteoglycans fine-tune mammalian physiology. Nature. (2007) 446:1030–7. 10.1038/nature05817 17460664

[93] Couchman JR Pataki CA. An introduction to proteoglycans and their localization. J Histochem Cytochem. (2012) 60:885–97. 10.1369/0022155412464638 23019015 PMC3527888

[94] Knox S Merry C Stringer S Melrose J Whitelock J. Not all perlecans are created equal: interactions with fibroblast growth factor (FGF) 2 and FGF receptors. J Biol Chem. (2002) 277:14657–65. 10.1074/jbc.M111826200 11847221

[95] Iozzo RV San Antonio JD. Heparan sulfate proteoglycans: heavy hitters in the angiogenesis arena. J Clin Invest. (2001) 108:349–55. 10.1172/JCI200113738 11489925 PMC209371

[96] Kim CW Goldberger OA Gallo RL Bernfield M. Members of the syndecan family of heparan sulfate proteoglycans are expressed in distinct cell-, tissue-, and development-specific patterns. Mol Biol Cell. (1994) 5:797–805. 10.1091/mbc.5.7.797 7812048 PMC301097

[97] Fransson LA Belting M Cheng F Jonsson M Mani K Sandgren S. Novel aspects of glypican glycobiology. Cell Mol Life Sci. (2004) 61:1016–24. 10.1007/s00018-004-3445-0 15112050 PMC11138644

[98] Manon-Jensen T Itoh Y Couchman JR. Proteoglycans in health and disease: the multiple roles of syndecan shedding. FEBS J. (2010) 277:3876–89. 10.1111/j.1742-4658.2010.07798.x 20840585

[99] Theocharis AD Skandalis SS Tzanakakis GN Karamanos NK. Proteoglycans in health and disease: novel roles for proteoglycans in malignancy and their pharmacological targeting. FEBS J. (2010) 277:3904–23. 10.1111/j.1742-4658.2010.07800.x 20840587

[100] Capurro MI Xiang YY Lobe C Filmus J. Glypican-3 promotes the growth of hepatocellular carcinoma by stimulating canonical Wnt signaling. Cancer Res. (2005) 65:6245–54. 10.1158/0008-5472.CAN-04-4244 16024626

[101] Cheng W Tseng CJ Lin TT Cheng I Pan HW Hsu HC. Glypican-3-mediated oncogenesis involves the insulin-like growth factor-signaling pathway. Carcinogenesis. (2008) 29:1319–26. 10.1093/carcin/bgn091 18413366 PMC2500215

[102] Zittermann SI Capurro MI Shi W Filmus J. Soluble glypican 3 inhibits the growth of hepatocellular carcinoma *in vitro* and *in vivo*. Int J Cancer. (2010) 126:1291–301. 10.1002/ijc.24941 19816934

[103] Aikawa T Whipple CA Lopez ME Gunn J Young A Lander AD. Glypican-1 modulates the angiogenic and metastatic potential of human and mouse cancer cells. J Clin Invest. (2008) 118:89–99. 10.1172/JCI32412 18064304 PMC2117766

[104] Kleeff J Ishiwata T Kumbasar A Friess H Buchler MW Lander AD. The cell-surface heparan sulfate proteoglycan glypican-1 regulates growth factor action in pancreatic carcinoma cells and is overexpressed in human pancreatic cancer. J Clin Invest. (1998) 102:1662–73. 10.1172/JCI4105 9802880 PMC509114

[105] Bernabeu C Lopez-Novoa JM Quintanilla M. The emerging role of TGF-beta superfamily coreceptors in cancer. Biochim Biophys Acta. (2009) 1792:954–73. 10.1016/j.bbadis.2009.07.003 19607914

[106] Chen C Zhao S Karnad A Freeman JW. The biology and role of CD44 in cancer progression: therapeutic implications. J Hematol Oncol. (2018) 11:64. 10.1186/s13045-018-0605-5 29747682 PMC5946470

[107] Farach-Carson MC Warren CR Harrington DA Carson DD. Border patrol: insights into the unique role of perlecan/heparan sulfate proteoglycan 2 at cell and tissue borders. Matrix Biol. (2014) 34:64–79. 10.1016/j.matbio.2013.08.004 24001398 PMC3938997

[108] Sanderson RD. Heparan sulfate proteoglycans in invasion and metastasis. Semin Cell Dev Biol. (2001) 12:89–98. 10.1006/scdb.2000.0241 11292374

[109] Yu WH Woessner JF Jr. Heparan sulfate proteoglycans as extracellular docking molecules for matrilysin (matrix metalloproteinase 7). J Biol Chem. (2000) 275:4183–91. 10.1074/jbc.275.6.4183 10660581

[110] Kelly T Mueller SC Yeh Y Chen WT. Invadopodia promote proteolysis of a wide variety of extracellular matrix proteins. J Cell Physiol. (1994) 158:299–308. 10.1002/jcp.1041580212 8106567

[111] Kelly T Yan Y Osborne RL Athota AB Rozypal TL Colclasure JC. Proteolysis of extracellular matrix by invadopodia facilitates human breast cancer cell invasion and is mediated by matrix metalloproteinases. Clin Exp Metastasis. (1998) 16:501–12. 10.1023/A:1006538200886 9872598

[112] Nguyen M Arkell J Jackson CJ. Active and tissue inhibitor of matrix metalloproteinase-free gelatinase B accumulates within human microvascular endothelial vesicles. J Biol Chem. (1998) 273:5400–4. 10.1074/jbc.273.9.5400 9479001

[113] Kato M Wang H Kainulainen V Fitzgerald ML Ledbetter S Ornitz DM. Physiological degradation converts the soluble syndecan-1 ectodomain from an inhibitor to a potent activator of FGF-2. Nat Med. (1998) 4:691–7. 10.1038/nm0698-691 9623978

[114] Esko JD Lindahl U. Molecular diversity of heparan sulfate. J Clin Invest. (2001) 108:169–73. 10.1172/JCI200113530 11457867 PMC203033

[115] Zhang L Esko JD. Amino acid determinants that drive heparan sulfate assembly in a proteoglycan. J Biol Chem. (1994) 269:19295–9. 8034692

[116] Kreuger J Kjellen L. Heparan sulfate biosynthesis: regulation and variability. J Histochem Cytochem. (2012) 60:898–907. 10.1369/0022155412464972 23042481 PMC3527889

[117] Stauber DJ DiGabriele AD Hendrickson WA. Structural interactions of fibroblast growth factor receptor with its ligands. Proc Natl Acad Sci USA. (2000) 97:49–54. 10.1073/pnas.97.1.49 10618369 PMC26614

[118] Guimond S Maccarana M Olwin BB Lindahl U Rapraeger AC. Activating and inhibitory heparin sequences for FGF-2 (basic FGF). distinct requirements for FGF-1, FGF-2, and FGF-4. J Biol Chem. (1993) 268:23906–14. 7693696

[119] Sugaya N Habuchi H Nagai N Ashikari-Hada S Kimata K. 6-O-sulfation of heparan sulfate differentially regulates various fibroblast growth factor-dependent signalings in culture. J Biol Chem. (2008) 283:10366–76. 10.1074/jbc.M705948200 18281280

[120] Suhovskih AV Domanitskaya NV Tsidulko AY Prudnikova TY Kashuba VI Grigorieva EV. Tissue-specificity of heparan sulfate biosynthetic machinery in cancer. Cell Adh Migr. (2015) 9:452–9. 10.1080/19336918.2015.1049801 26120938 PMC4955958

[121] Weyers A Yang B Yoon DS Park JH Zhang F Lee KB. A structural analysis of glycosaminoglycans from lethal and nonlethal breast cancer tissues: toward a novel class of theragnostics for personalized medicine in oncology? OMICS. (2012) 16:79–89. 10.1089/omi.2011.0102 22401653 PMC3300064

[122] Jayson GC Lyon M Paraskeva C Turnbull JE Deakin JA Gallagher JT. Heparan sulfate undergoes specific structural changes during the progression from human colon adenoma to carcinoma *in vitro*. J Biol Chem. (1998) 273:51–7. 10.1074/jbc.273.1.51 9417046

[123] Rangel MP de Sa VK Prieto T Martins JRM Olivieri ER Carraro D. Biomolecular analysis of matrix proteoglycans as biomarkers in non small cell lung cancer. Glycoconj J. (2018) 35:233–42. 10.1007/s10719-018-9815-x 29502190

[124] Fernandez-Vega I Garcia O Crespo A Castanon S Menendez P Astudillo A. Specific genes involved in synthesis and editing of heparan sulfate proteoglycans show altered expression patterns in breast cancer. BMC Cancer. (2013) 13:24. 10.1186/1471-2407-13-24 23327652 PMC3561094

[125] Nagamine S Tamba M Ishimine H Araki K Shiomi K Okada T. Organ-specific sulfation patterns of heparan sulfate generated by extracellular sulfatases Sulf1 and Sulf2 in mice. J Biol Chem. (2012) 287:9579–90. 10.1074/jbc.M111.290262 22298771 PMC3308746

[126] Khurana A Jung-Beom D He X Kim SH Busby RC Lorenzon L. Matrix detachment and proteasomal inhibitors diminish Sulf-2 expression in breast cancer cell lines and mouse xenografts. Clin Exp Metastasis. (2013) 30:407–15. 10.1007/s10585-012-9546-5 23412907 PMC3619208

[127] Morimoto-Tomita M Uchimura K Werb Z Hemmerich S Rosen SD. Cloning and characterization of two extracellular heparin-degrading endosulfatases in mice and humans. J Biol Chem. (2002) 277:49175–85. 10.1074/jbc.M205131200 12368295 PMC2779716

[128] Lai JP Sandhu DS Yu C Han T Moser CD Jackson KK. Sulfatase 2 up-regulates glypican 3, promotes fibroblast growth factor signaling, and decreases survival in hepatocellular carcinoma. Hepatology. (2008) 47:1211–22. 10.1002/hep.22202 18318435 PMC2536494

[129] Lai JP Sandhu DS Shire AM Roberts LR. The tumor suppressor function of human sulfatase 1 (SULF1) in carcinogenesis. J Gastrointest Cancer. (2008) 39:149–58. 10.1007/s12029-009-9058-y 19373441 PMC2925118

[130] Phillips JJ Huillard E Robinson AE Ward A Lum DH Polley MY. Heparan sulfate sulfatase SULF2 regulates PDGFRalpha signaling and growth in human and mouse malignant glioma. J Clin Invest. (2012) 122:911–22. 10.1172/JCI58215 22293178 PMC3287216

[131] Rosen SD Lemjabbar-Alaoui H. Sulf-2: an extracellular modulator of cell signaling and a cancer target candidate. Expert Opin Ther Targets. (2010) 14:935–49. 10.1517/14728222.2010.504718 20629619 PMC3126665

[132] Vicente CM Lima MA Nader HB Toma L. SULF2 overexpression positively regulates tumorigenicity of human prostate cancer cells. J Exp Clin Cancer Res. (2015) 34:25. 10.1186/s13046-015-0141-x 25887999 PMC4374423

[133] Thiery JP Acloque H Huang RY Nieto MA. Epithelial-mesenchymal transitions in development and disease. Cell. (2009) 139:871–90. 10.1016/j.cell.2009.11.007 19945376

[134] Polyak K Weinberg RA. Transitions between epithelial and mesenchymal states: acquisition of malignant and stem cell traits. Nat Rev Cancer. (2009) 9:265–73. 10.1038/nrc2620 19262571

[135] Maupin KA Sinha A Eugster E Miller J Ross J Paulino V. Glycogene expression alterations associated with pancreatic cancer epithelial-mesenchymal transition in complementary model systems. PLoS ONE. (2010) 5:e13002. 10.1371/journal.pone.0013002 20885998 PMC2946336

[136] Chen Z Fan JQ Li J Li QS Yan Z Jia XK. Promoter hypermethylation correlates with the Hsulf-1 silencing in human breast and gastric cancer. Int J Cancer. (2009) 124:739–44. 10.1002/ijc.23960 19006069

[137] Damiens E El Yazidi I Mazurier J Elass-Rochard E Duthille I Spik G. Role of heparan sulphate proteoglycans in the regulation of human lactoferrin binding and activity in the MDA-MB-231 breast cancer cell line. Eur J Cell Biol. (1998) 77:344–51. 10.1016/S0171-9335(98)80093-9 9930659

[138] Garcia-Suarez O Garcia B Fernandez-Vega I Astudillo A Quiros LM. Neuroendocrine tumors show altered expression of chondroitin sulfate, glypican 1, glypican 5, and syndecan 2 depending on their differentiation grade. Front Oncol. (2014) 4:15. 10.3389/fonc.2014.00015 24570896 PMC3917325

[139] Bloushtain N Qimron U Bar-Ilan A Hershkovitz O Gazit R Fima E. Membrane-associated heparan sulfate proteoglycans are involved in the recognition of cellular targets by NKp30 and NKp46. J Immunol. (2004) 173:2392–401. 10.4049/jimmunol.173.4.2392 15294952

[140] Gubbiotti MA Neill T Iozzo RV. A current view of perlecan in physiology and pathology: a mosaic of functions. Matrix Biol. (2017) 57–8:285–98. 10.1016/j.matbio.2016.09.003 27613501 PMC5328851

[141] Douglass S Goyal A Iozzo RV. The role of perlecan and endorepellin in the control of tumor angiogenesis and endothelial cell autophagy. Connect Tissue Res. (2015) 56:381–91. 10.3109/03008207.2015.1045297 26181327 PMC4769797

[142] Whitelock JM Melrose J Iozzo RV. Diverse cell signaling events modulated by perlecan. Biochemistry. (2008) 47:11174–83. 10.1021/bi8013938 18826258 PMC2605657

[143] Segev A Nili N Strauss BH. The role of perlecan in arterial injury and angiogenesis. Cardiovasc Res. (2004) 63:603–10. 10.1016/j.cardiores.2004.03.028 15306215

[144] Isemura M Sato N Yamaguchi Y Aikawa J Munakata H Hayashi N. Isolation and characterization of fibronectin-binding proteoglycan carrying both heparan sulfate and dermatan sulfate chains from human placenta. J Biol Chem. (1987) 262:8926–33. 2954956

[145] Couchman JR Kapoor R Sthanam M Wu RR. Perlecan and basement membrane-chondroitin sulfate proteoglycan (bamacan) are two basement membrane chondroitin/dermatan sulfate proteoglycans in the Engelbreth-Holm-Swarm tumor matrix. J Biol Chem. (1996) 271:9595–602. 10.1074/jbc.271.16.9595 8621634

[146] Brown JC Sasaki T Gohring W Yamada Y Timpl R. The C-terminal domain V of perlecan promotes beta1 integrin-mediated cell adhesion, binds heparin, nidogen and fibulin-2 and can be modified by glycosaminoglycans. Eur J Biochem. (1997) 250:39–46. 10.1111/j.1432-1033.1997.t01-1-00039.x 9431988

[147] Mongiat M Taylor K Otto J Aho S Uitto J Whitelock JM. The protein core of the proteoglycan perlecan binds specifically to fibroblast growth factor-7. J Biol Chem. (2000) 275:7095–100. 10.1074/jbc.275.10.7095 10702276

[148] Gohring W Sasaki T Heldin CH Timpl R. Mapping of the binding of platelet-derived growth factor to distinct domains of the basement membrane proteins BM-40 and perlecan and distinction from the BM-40 collagen-binding epitope. Eur J Biochem. (1998) 255:60–6. 10.1046/j.1432-1327.1998.2550060.x 9692901

[149] Woodall BP Nystrom A Iozzo RA Eble JA Niland S Krieg T. Integrin alpha2beta1 is the required receptor for endorepellin angiostatic activity. J Biol Chem. (2008) 283:2335–43. 10.1074/jbc.M708364200 18024432

[150] Cailhier JF Sirois I Laplante P Lepage S Raymond MA Brassard N. Caspase-3 activation triggers extracellular cathepsin L release and endorepellin proteolysis. J Biol Chem. (2008) 283:27220–9. 10.1074/jbc.M801164200 18658137

[151] Murdoch AD Dodge GR Cohen I Tuan RS Iozzo RV. Primary structure of the human heparan sulfate proteoglycan from basement membrane (HSPG2/perlecan). A chimeric molecule with multiple domains homologous to the low density lipoprotein receptor, laminin, neural cell adhesion molecules, and epidermal growth factor. J Biol Chem. (1992) 267:8544–57. 1569102

[152] Dolan M Horchar T Rigatti B Hassell JR. Identification of sites in domain I of perlecan that regulate heparan sulfate synthesis. J Biol Chem. (1997) 272:4316–22. 10.1074/jbc.272.7.4316 9020150

[153] Zhou Z Wang J Cao R Morita H Soininen R Chan KM. Impaired angiogenesis, delayed wound healing and retarded tumor growth in perlecan heparan sulfate-deficient mice. Cancer Res. (2004) 64:4699–702. 10.1158/0008-5472.CAN-04-0810 15256433

[154] Jiang X Couchman JR. Perlecan and tumor angiogenesis. J Histochem Cytochem. (2003) 51:1393–410. 10.1177/002215540305101101 14566013 PMC3957550

[155] Ornitz DM. FGFs, heparan sulfate and FGFRs: complex interactions essential for development. Bioessays. (2000) 22:108–12. 10.1002/(SICI)1521-1878(200002)22:2<108::AID-BIES2>3.0.CO;2-M 10655030

[156] Aviezer D Hecht D Safran M Eisinger M David G Yayon A. Perlecan, basal lamina proteoglycan, promotes basic fibroblast growth factor-receptor binding, mitogenesis, and angiogenesis. Cell. (1994) 79:1005–13. 10.1016/0092-8674(94)90031-0 7528102

[157] Lord MS Chuang CY Melrose J Davies MJ Iozzo RV Whitelock JM. The role of vascular-derived perlecan in modulating cell adhesion, proliferation and growth factor signaling. Matrix Biol. (2014) 35:112–22. 10.1016/j.matbio.2014.01.016 24509440 PMC5030467

[158] Goyal A Pal N Concannon M Paul M Doran M Poluzzi C. Endorepellin, the angiostatic module of perlecan, interacts with both the alpha2beta1 integrin and vascular endothelial growth factor receptor 2 (VEGFR2): a dual receptor antagonism. J Biol Chem. (2011) 286:25947–62. 10.1074/jbc.M111.243626 21596751 PMC3138248

[159] Goyal A Poluzzi C Willis CD Smythies J Shellard A Neill T. Endorepellin affects angiogenesis by antagonizing diverse vascular endothelial growth factor receptor 2 (VEGFR2)-evoked signaling pathways: transcriptional repression of hypoxia-inducible factor 1alpha and VEGFA and concurrent inhibition of nuclear factor of activated T cell 1 (NFAT1) activation. J Biol Chem. (2012) 287:43543–56. 10.1074/jbc.M112.401786 23060442 PMC3527941

[160] Willis CD Poluzzi C Mongiat M Iozzo RV. Endorepellin laminin-like globular 1/2 domains bind Ig3-5 of vascular endothelial growth factor (VEGF) receptor 2 and block pro-angiogenic signaling by VEGFA in endothelial cells. FEBS J. (2013) 280:2271–84. 10.1111/febs.12164 23374253 PMC3651768

[161] Bix G Fu J Gonzalez EM Macro L Barker A Campbell S. Endorepellin causes endothelial cell disassembly of actin cytoskeleton and focal adhesions through alpha2beta1 integrin. J Cell Biol. (2004) 166:97–109. 10.1083/jcb.200401150 15240572 PMC2172143

[162] Gonzalez EM Reed CC Bix G Fu J Zhang Y Gopalakrishnan B. BMP-1/Tolloid-like metalloproteases process endorepellin, the angiostatic C-terminal fragment of perlecan. J Biol Chem. (2005) 280:7080–7. 10.1074/jbc.M409841200 15591058

[163] Chang JW Kang UB Kim DH Yi JK Lee JW Noh DY. Identification of circulating endorepellin LG3 fragment: potential use as a serological biomarker for breast cancer. Proteomics Clin Appl. (2008) 2:23–32. 10.1002/prca.200780049 21136776

[164] Cohen IR Murdoch AD Naso MF Marchetti D Berd D Iozzo RV. Abnormal expression of perlecan proteoglycan in metastatic melanomas. Cancer Res. (1994) 54:5771–4. 7954396

[165] Ilhan-Mutlu A Siehs C Berghoff AS Ricken G Widhalm G Wagner L. Expression profiling of angiogenesis-related genes in brain metastases of lung cancer and melanoma. Tumour Biol. (2016) 37:1173–82. 10.1007/s13277-015-3790-7 26277786

[166] Iozzo RV Cohen I. Altered proteoglycan gene expression and the tumor stroma. EXS. (1994) 70:199–214. 10.1007/978-3-0348-7545-5_12 8298247

[167] Nackaerts K Verbeken E Deneffe G Vanderschueren B Demedts M David G. Heparan sulfate proteoglycan expression in human lung-cancer cells. Int J Cancer. (1997) 74:335–45. 10.1002/(SICI)1097-0215(19970620)74:3<335::AID-IJC18>3.0.CO;2-A 9221815

[168] Nerlich AG Wiest I Wagner E Sauer U Schleicher ED. Gene expression and protein deposition of major basement membrane components and TGF-beta 1 in human breast cancer. Anticancer Res. (1997) 17:4443–9. 9494547

[169] Nerlich AG Lebeau A Hagedorn HG Sauer U Schleicher ED. Morphological aspects of altered basement membrane metabolism in invasive carcinomas of the breast and the larynx. Anticancer Res. (1998) 18:3515–20. 9858933

[170] Roskams T De Vos R David G Van Damme B Desmet V. Heparan sulphate proteoglycan expression in human primary liver tumours. J Pathol. (1998) 185:290–7. 10.1002/(SICI)1096-9896(199807)185:3<290::AID-PATH91>3.0.CO;2-I 9771483

[171] Sabit H Tsuneyama K Shimonishi T Harada K Cheng J Ida H. Enhanced expression of basement-membrane-type heparan sulfate proteoglycan in tumor fibro-myxoid stroma of intrahepatic cholangiocarcinoma. Pathol Int. (2001) 51:248–56. 10.1046/j.1440-1827.2001.01201.x 11350606

[172] Ida-Yonemochi H Ikarashi T Nagata M Hoshina H Takagi R Saku T. The basement membrane-type heparan sulfate proteoglycan (perlecan) in ameloblastomas: its intercellular localization in stellate reticulum-like foci and biosynthesis by tumor cells in culture. Virchows Arch. (2002) 441:165–73. 10.1007/s00428-001-0556-y 12189507

[173] Warren CR Grindel BJ Francis L Carson DD Farach-Carson MC. Transcriptional activation by NFkappaB increases perlecan/HSPG2 expression in the desmoplastic prostate tumor microenvironment. J Cell Biochem. (2014) 115:1322–33. 10.1002/jcb.24788 24700612 PMC4091977

[174] Davies G Cunnick GH Mansel RE Mason MD Jiang WG. Levels of expression of endothelial markers specific to tumour-associated endothelial cells and their correlation with prognosis in patients with breast cancer. Clin Exp Metastasis. (2004) 21:31–7. 10.1023/B:CLIN.0000017168.83616.d0 15065600

[175] Gronborg M Kristiansen TZ Iwahori A Chang R Reddy R Sato N. Biomarker discovery from pancreatic cancer secretome using a differential proteomic approach. Mol Cell Proteomics. (2006) 5:157–71. 10.1074/mcp.M500178-MCP200 16215274

[176] Hasegawa M Cheng J Maruyama S Yamazaki M Abe T Babkair H. Differential immunohistochemical expression profiles of perlecan-binding growth factors in epithelial dysplasia, carcinoma *in situ*, and squamous cell carcinoma of the oral mucosa. Pathol Res Pract. (2016) 212:426–36. 10.1016/j.prp.2016.02.016 26965914

[177] Kazanskaya GM Tsidulko AY Volkov AM Kiselev RS Suhovskih AV Kobozev VV. Heparan sulfate accumulation and perlecan/HSPG2 up-regulation in tumour tissue predict low relapse-free survival for patients with glioblastoma. Histochem Cell Biol. (2018) 149:235–44. 10.1007/s00418-018-1631-7 29322326

[178] Hynes RO. The extracellular matrix: not just pretty fibrils. Science. (2009) 326:1216–19. 10.1126/science.1176009 19965464 PMC3536535

[179] Kalluri R. Basement membranes: structure, assembly and role in tumour angiogenesis. Nat Rev Cancer. (2003) 3:422–33. 10.1038/nrc1094 12778132

[180] Walker C Mojares E Del Rio Hernandez A. Role of extracellular matrix in development and cancer progression. Int J Mol Sci. (2018) 19:E3028. 10.3390/ijms19103028 30287763 PMC6213383

[181] Humphrey JD Dufresne ER Schwartz MA. Mechanotransduction and extracellular matrix homeostasis. Nat Rev Mol Cell Biol. (2014) 15:802–12. 10.1038/nrm3896 25355505 PMC4513363

[182] Cruz-Munoz W Khokha R. The role of tissue inhibitors of metalloproteinases in tumorigenesis and metastasis. Crit Rev Clin Lab Sci. (2008) 45:291–338. 10.1080/10408360801973244 18568853

[183] Troeberg L Lazenbatt CEK Anower MF Freeman C Federov O Habuchi H. Sulfated glycosaminoglycans control the extracellular trafficking and the activity of the metalloprotease inhibitor TIMP-3. Chem Biol. (2014) 21:1300–9. 10.1016/j.chembiol.2014.07.014 25176127 PMC4210636

[184] Malik R Lelkes PI Cukierman E. Biomechanical and biochemical remodeling of stromal extracellular matrix in cancer. Trends Biotechnol. (2015) 33:230–6. 10.1016/j.tibtech.2015.01.004 25708906 PMC4380578

[185] Bonnans C Chou J Werb Z. Remodelling the extracellular matrix in development and disease. Nat Rev Mol Cell Biol. (2014) 15:786–801. 10.1038/nrm3904 25415508 PMC4316204

[186] Glentis A Gurchenkov V Matic Vignjevic D. Assembly, heterogeneity, and breaching of the basement membranes. Cell Adh Migr. (2014) 8:236–45. 10.4161/cam.28733 24727304 PMC4198347

[187] Lu P Takai K Weaver VM Werb Z. Extracellular matrix degradation and remodeling in development and disease. Cold Spring Harb Perspect Biol. (2011) 3:a005058. 10.1101/cshperspect.a005058 21917992 PMC3225943

[188] De Palma M Biziato D Petrova TV. Microenvironmental regulation of tumour angiogenesis. Nat Rev Cancer. (2017) 17:457–74. 10.1038/nrc.2017.51 28706266

[189] Fuster MM Wang L Castagnola J Sikora L Reddi K Lee PH. Genetic alteration of endothelial heparan sulfate selectively inhibits tumor angiogenesis. J Cell Biol. (2007) 177:539–49. 10.1083/jcb.200610086 17470635 PMC2064806

[190] Jiang X Multhaupt H Chan E Schaefer L Schaefer RM Couchman JR. Essential contribution of tumor-derived perlecan to epidermal tumor growth and angiogenesis. J Histochem Cytochem. (2004) 52:1575–90. 10.1369/jhc.4A6353.2004 15557212

[191] Li H Fan X Houghton J. Tumor microenvironment: the role of the tumor stroma in cancer. J Cell Biochem. (2007) 101:805–15. 10.1002/jcb.21159 17226777

[192] Carmeliet P. VEGF as a key mediator of angiogenesis in cancer. Oncology. (2005) 69 (Suppl 3):4–10. 10.1159/000088478 16301830

[193] Savore C Zhang C Muir C Liu R Wyrwa J Shu J. Perlecan knockdown in metastatic prostate cancer cells reduces heparin-binding growth factor responses *in vitro* and tumor growth *in vivo*. Clin Exp Metastasis. (2005) 22:377–90. 10.1007/s10585-005-2339-3 16283481

[194] Minchenko A Bauer T Salceda S Caro J. Hypoxic stimulation of vascular endothelial growth factor expression *in vitro* and *in vivo*. Lab Invest. (1994) 71:374–9. 7933988

[195] Li J Shworak NW Simons M. Increased responsiveness of hypoxic endothelial cells to FGF2 is mediated by HIF-1alpha-dependent regulation of enzymes involved in synthesis of heparan sulfate FGF2-binding sites. J Cell Sci. (2002) 115(Pt 9):1951–9. Available online at: https://jcs.biologists.org/content/115/9/1951 11956326 10.1242/jcs.115.9.1951

[196] Vlodavsky I Beckhove P Lerner I Pisano C Meirovitz A Ilan N. Significance of heparanase in cancer and inflammation. Cancer Microenviron. (2012) 5:115–32. 10.1007/s12307-011-0082-7 21811836 PMC3399068

[197] Iozzo RV Zoeller JJ Nystrom A. Basement membrane proteoglycans: modulators Par Excellence of cancer growth and angiogenesis. Mol Cells. (2009) 27:503–13. 10.1007/s10059-009-0069-0 19466598 PMC6712562

[198] Pikas DS Li JP Vlodavsky I Lindahl U. Substrate specificity of heparanases from human hepatoma and platelets. J Biol Chem. (1998) 273:18770–7. 10.1074/jbc.273.30.18770 9668050

[199] Roy M Marchetti D. Cell surface heparan sulfate released by heparanase promotes melanoma cell migration and angiogenesis. J Cell Biochem. (2009) 106:200–9. 10.1002/jcb.22005 19115257 PMC2736788

[200] Vlodavsky I Gross-Cohen M Weissmann M Ilan N Sanderson RD. Opposing functions of heparanase-1 and heparanase-2 in cancer progression. Trends Biochem Sci. (2018) 43:18–31. 10.1016/j.tibs.2017.10.007 29162390 PMC5741533

[201] Masola V Bellin G Gambaro G Onisto M. Heparanase: a multitasking protein involved in extracellular matrix (ECM) remodeling and intracellular events. Cells. (2018) 7:E236. 10.3390/cells7120236 30487472 PMC6316874

[202] Arvatz G Weissmann M Ilan N Vlodavsky I. Heparanase and cancer progression: new directions, new promises. Hum Vaccin Immunother. (2016) 12:2253–6. 10.1080/21645515.2016.1171442 27054564 PMC5027699

[203] Sanderson RD Elkin M Rapraeger AC Ilan N Vlodavsky I. Heparanase regulation of cancer, autophagy and inflammation: new mechanisms and targets for therapy. FEBS J. (2017) 284:42–55. 10.1111/febs.13932 27758044 PMC5226874

[204] Murry BP Greiter-Wilke A Paulsen DP Hiatt KM Beltrami CA Marchetti D. Selective heparanase localization in malignant melanoma. Int J Oncol. (2005) 26:345–52. 10.3892/ijo.26.2.345 15645118

[205] Komatsu N Waki M Sue M Tokuda C Kasaoka T Nakajima M. Heparanase expression in B16 melanoma cells and peripheral blood neutrophils before and after extravasation detected by novel anti-mouse heparanase monoclonal antibodies. J Immunol Methods. (2008) 331:82–93. 10.1016/j.jim.2007.11.014 18162185

[206] Cohen I Pappo O Elkin M San T Bar-Shavit R Hazan R. Heparanase promotes growth, angiogenesis and survival of primary breast tumors. Int J Cancer. (2006) 118:1609–17. 10.1002/ijc.21552 16217746

[207] Friedmann Y Vlodavsky I Aingorn H Aviv A Peretz T Pecker I. Expression of heparanase in normal, dysplastic, and neoplastic human colonic mucosa and stroma. Evidence for its role in colonic tumorigenesis. Am J Pathol. (2000) 157:1167–75. 10.1016/S0002-9440(10)64632-9 11021821 PMC1850180

[208] Murry BP Blust BE Singh A Foster TP Marchetti D. Heparanase mechanisms of melanoma metastasis to the brain: Development and use of a brain slice model. J Cell Biochem. (2006) 97:217–25. 10.1002/jcb.20714 16288472

[209] Marchetti D Nicolson GL. Human heparanase: a molecular determinant of brain metastasis. Adv Enzyme Regul. (2001) 41:343–59. 10.1016/S0065-2571(00)00016-9 11384754

[210] Theodoro TR de Matos LL Sant Anna AV Fonseca FL Semedo P Martins LC Heparanase expression in circulating lymphocytes of breast cancer patients depends on the presence of the primary tumor and/or systemic metastasis. Neoplasia. (2007) 9:504–10. 10.1593/neo.07241 17603633 PMC1899258

[211] Vlodavsky I Friedmann Y Elkin M Aingorn H Atzmon R Ishai-Michaeli R. Mammalian heparanase: gene cloning, expression and function in tumor progression and metastasis. Nat Med. (1999) 5:793–802. 10.1038/10518 10395325

[212] He X Brenchley PE Jayson GC Hampson L Davies J Hampson IN. Hypoxia increases heparanase-dependent tumor cell invasion, which can be inhibited by antiheparanase antibodies. Cancer Res. (2004) 64:3928–33. 10.1158/0008-5472.CAN-03-2718 15173004

[213] Jingting C Yangde Z Yi Z Huining L Rong Y Yu Z. Heparanase expression correlates with metastatic capability in human choriocarcinoma. Gynecol Oncol. (2007) 107:22–9. 10.1016/j.ygyno.2007.05.042 17688924

[214] Liu D Shriver Z Venkataraman G El Shabrawi Y Sasisekharan R Tumor cell surface heparan sulfate as cryptic promoters or inhibitors of tumor growth and metastasis. Proc Natl Acad Sci USA. (2002) 99:568–73. 10.1073/pnas.012578299 11805315 PMC117346

[215] Fux L Ilan N Sanderson RD Vlodavsky I. Heparanase: busy at the cell surface. Trends Biochem Sci. (2009) 34:511–9. 10.1016/j.tibs.2009.06.005 19733083 PMC2755511

[216] Furuta J Umebayashi Y Miyamoto K Kikuchi K Otsuka F Sugimura T. Promoter methylation profiling of 30 genes in human malignant melanoma. Cancer Sci. (2004) 95:962–8. 10.1111/j.1349-7006.2004.tb03184.x 15596045 PMC11160084

[217] Ma YQ Geng JG. Heparan sulfate-like proteoglycans mediate adhesion of human malignant melanoma A375 cells to P-selectin under flow. J Immunol. (2000) 165:558–65. 10.4049/jimmunol.165.1.558 10861096

[218] Jung O Trapp-Stamborski V Purushothaman A Jin H Wang H Sanderson RD. Heparanase-induced shedding of syndecan-1/CD138 in myeloma and endothelial cells activates VEGFR2 and an invasive phenotype: prevention by novel synstatins. Oncogenesis. (2016) 5:e202. 10.1038/oncsis.2016.5 26926788 PMC5154350

[219] Rowe RG Weiss SJ. Breaching the basement membrane: who, when and how? Trends Cell Biol. (2008) 18:560–74. 10.1016/j.tcb.2008.08.007 18848450

[220] Murdoch AD Liu B Schwarting R Tuan RS Iozzo RV. Widespread expression of perlecan proteoglycan in basement membranes and extracellular matrices of human tissues as detected by a novel monoclonal antibody against domain III and by *in situ* hybridization. J Histochem Cytochem. (1994) 42:239–49. 10.1177/42.2.7507142 7507142

[221] Klein G Conzelmann S Beck S Timpl R Muller CA. Perlecan in human bone marrow: a growth-factor-presenting, but anti-adhesive, extracellular matrix component for hematopoietic cells. Matrix Biol. (1995) 14:457–65. 10.1016/0945-053X(95)90003-9 7795884

[222] Grindel BJ Martinez JR Pennington CL Muldoon M Stave J Chung LW. Matrilysin/matrix metalloproteinase-7(MMP7) cleavage of perlecan/HSPG2 creates a molecular switch to alter prostate cancer cell behavior. Matrix Biol. (2014) 36:64–76. 10.1016/j.matbio.2014.04.005 24833109 PMC4748839

[223] Grindel BJ Martinez JR Tellman TV Harrington DA Zafar H Nakhleh L. Matrilysin/MMP-7 cleavage of perlecan/HSPG2 complexed with semaphorin 3A supports FAK-mediated stromal invasion by prostate cancer cells. Sci Rep. (2018) 8:7262. 10.1038/s41598-018-25435-3 29740048 PMC5940808

[224] Marchetti D Menter D Jin L Nakajima M Nicolson GL. Nerve growth factor effects on human and mouse melanoma cell invasion and heparanase production. Int J Cancer. (1993) 55:692–9. 10.1002/ijc.2910550430 8407001

[225] Sharma B Handler M Eichstetter I Whitelock JM Nugent MA Iozzo RV. Antisense targeting of perlecan blocks tumor growth and angiogenesis *in vivo*. J Clin Invest. (1998) 102:1599–608. 10.1172/JCI3793 9788974 PMC509011

[226] Adatia R Albini A Carlone S Giunciuglio D Benelli R Santi L. Suppression of invasive behavior of melanoma cells by stable expression of anti-sense perlecan cDNA. Ann Oncol. (1997) 8:1257–61. 10.1023/A:1008243115385 9496392

[227] Mathiak M Yenisey C Grant DS Sharma B Iozzo RV. A role for perlecan in the suppression of growth and invasion in fibrosarcoma cells. Cancer Res. (1997) 57:2130–6. 9187109

[228] Datta S Pierce M Datta MW. Perlecan signaling: helping hedgehog stimulate prostate cancer growth. Int J Biochem Cell Biol. (2006) 38:1855–61. 10.1016/j.biocel.2006.03.022 16750652

[229] Metwaly H Maruyama S Yamazaki M Tsuneki M Abe T Jen KY. Parenchymal-stromal switching for extracellular matrix production on invasion of oral squamous cell carcinoma. Hum Pathol. (2012) 43:1973–81. 10.1016/j.humpath.2012.02.006 22575259

[230] Maruyama S Shimazu Y Kudo T Sato K Yamazaki M Abe T. Three-dimensional visualization of perlecan-rich neoplastic stroma induced concurrently with the invasion of oral squamous cell carcinoma. J Oral Pathol Med. (2014) 43:627–36. 10.1111/jop.12184 24697873

[231] Datta MW Hernandez AM Schlicht MJ Kahler AJ DeGueme AM Dhir R. Perlecan, a candidate gene for the CAPB locus, regulates prostate cancer cell growth via the Sonic Hedgehog pathway. Mol Cancer. (2006) 5:9. 10.1186/1476-4598-5-9 16507112 PMC1421430

[232] Galli M Chatterjee M Grasso M Specchia G Magen H Einsele H Phase I study of the heparanase inhibitor roneparstat: an innovative approach for multiple myeloma therapy. Haematologica. (2018) 103:e469–72. 10.3324/haematol.2017.182865 29700168 PMC6165822

[233] Basche M Gustafson DL Holden SN O'Bryant CL Gore L Witta S. A phase I biological and pharmacologic study of the heparanase inhibitor PI-88 in patients with advanced solid tumors. Clin Cancer Res. (2006) 12:5471–80. 10.1158/1078-0432.CCR-05-2423 17000682

[234] O'Reilly EM Roach J Miller P Yu KH Tjan C Rosano M. Safety, pharmacokinetics, pharmacodynamics, and antitumor activity of necuparanib combined with Nab-Paclitaxel and gemcitabine in patients with metastatic pancreatic cancer: phase I results. Oncologist. (2017) 22:1429–e139. 10.1634/theoncologist.2017-0472 29158367 PMC5728039

[235] Dredge K Hammond E Handley P Gonda TJ Smith MT Vincent C. PG545, a dual heparanase and angiogenesis inhibitor, induces potent anti-tumour and anti-metastatic efficacy in preclinical models. Br J Cancer. (2011) 104:635. 10.1038/bjc.2011.11 21285983 PMC3049593

[236] Hammond E Brandt R Dredge K. PG545, a heparan sulfate mimetic, reduces heparanase expression *in vivo*, blocks spontaneous metastases and enhances overall survival in the 4T1 breast carcinoma model. PLoS ONE. (2012) 7:e52175. 10.1371/journal.pone.0052175 23300607 PMC3530599

[237] Katz A Barash U Boyango I Feld S Zohar Y Hammond E. Patient derived xenografts (PDX) predict an effective heparanase based therapy for lung cancer. Oncotarget. (2018) 9:19294–306. 10.18632/oncotarget.25022 29721203 PMC5922397

[238] MacDonald A Priess M Curran J Guess J Farutin V Oosterom I. Necuparanib, a multitargeting heparan sulfate mimetic, targets tumor and stromal compartments in pancreatic cancer. J Molecular Cancer Therapeutics. (2019) 18:245–56. 10.1158/1535-7163.MCT-18-0417 30401693

[239] Xia C Yin S Xu S Ran G Deng M Mei L. Low molecular weight heparin-coated and dendrimer-based core-shell nanoplatform with enhanced immune activation and multiple anti-metastatic effects for melanoma treatment. Theranostics. (2019) 9:337–54. 10.7150/thno.29026 30809278 PMC6376190

[240] Messore A Madia VN Pescatori L Saccoliti F Tudino V De Leo A. Novel symmetrical benzazolyl derivatives endowed with potent anti-heparanase activity. J Med Chem. (2018) 61:10834–59. 10.1021/acs.jmedchem.8b01497 30412404

[241] Zheng X Gai X Han S Moser CD Hu C Shire AM. The human sulfatase 2 inhibitor 2,4-disulfonylphenyl-tert-butylnitrone (OKN-007) has an antitumor effect in hepatocellular carcinoma mediated via suppression of TGFB1/SMAD2 and Hedgehog/GLI1 signaling. Genes Chromosome Cancer. (2013) 52:225–36. 10.1002/gcc.22022 23109092 PMC3889201

[242] Coutinho de Souza P Mallory S Smith N Saunders D Li X-N McNall-Knapp RY. Inhibition of pediatric glioblastoma tumor growth by the anti-cancer agent OKN-007 in orthotopic mouse xenografts. PLoS ONE. (2015) 10:e0134276. 10.1371/journal.pone.0134276 26248280 PMC4527837

[243] Bosse KR Raman P Zhu Z Lane M Martinez D Heitzeneder S. Identification of GPC2 as an oncoprotein and candidate immunotherapeutic target in high-risk neuroblastoma. Cancer Cell. (2017) 32:295–309.e12. 10.1016/j.ccell.2017.08.003 28898695 PMC5600520

[244] Zhu AX Gold PJ El-Khoueiry AB Abrams TA Morikawa H Ohishi N. First-in-man phase I study of GC33, a novel recombinant humanized antibody against glypican-3, in patients with advanced hepatocellular carcinoma. Clin Cancer Res. (2013) 19:920–8. 10.1158/1078-0432.CCR-12-2616 23362325

[245] Li W Guo L Rathi P Marinova E Gao X Wu MF. Redirecting T cells to glypican-3 with 4-1BB zeta chimeric antigen receptors results in Th1 polarization and potent antitumor activity. Hum Gene Ther. (2017) 28:437–48. 10.1089/hum.2016.025 27530312 PMC5444493

[246] Gao H Li K Tu H Pan X Jiang H Shi B. Development of T cells redirected to glypican-3 for the treatment of hepatocellular carcinoma. Clin Cancer Res. (2014) 20:6418–28. 10.1158/1078-0432.CCR-14-1170 25320357

[247] Lanzi C Zaffaroni N Cassinelli G. Targeting heparan sulfate proteoglycans and their modifying enzymes to enhance anticancer chemotherapy efficacy and overcome drug resistance. Curr Med Chem. (2017) 24:2860–86. 10.2174/0929867324666170216114248 28215163

[248] Lanzi C Cassinelli G. Heparan sulfate mimetics in cancer therapy: the challenge to define structural determinants and the relevance of targets for optimal activity. Molecules. (2018) 23:E2915. 10.3390/molecules23112915 30413079 PMC6278363

[249] Smorenburg SM. The effects of unfractionated heparin on survival in patients with malignancy - A systematic review. Thromb Haemost. (1999) 82:1600–4. 10.1055/s-0037-1614885 10613641

[250] Naggi A Casu B Perez M Torri G Cassinelli G Penco S. Modulation of the heparanase-inhibiting activity of heparin through selective desulfation, graded N-acetylation, and glycol splitting. J Biol Chem. (2005) 280:12103–13. 10.1074/jbc.M414217200 15647251

[251] Dredge K Brennan TV Hammond E Lickliter JD Lin L Bampton D. A Phase I study of the novel immunomodulatory agent PG545 (pixatimod) in subjects with advanced solid tumours. Br J Cancer. (2018) 118:1035–41. 10.1038/s41416-018-0006-0 29531325 PMC5931096

[252] Loka RS Sletten ET Barash U Vlodavsky I Nguyen HM. Specific inhibition of heparanase by a glycopolymer with well-defined sulfation pattern prevents breast cancer metastasis in mice. ACS Appl Mater Interfaces. (2019) 11:244–54. 10.1021/acsami.8b17625 30543095 PMC6512314

[253] Loka RS Yu F Sletten ET Nguyen HM. Design, synthesis, and evaluation of heparan sulfate mimicking glycopolymers for inhibiting heparanase activity. Chem Commun. (2017) 53:9163–66. 10.1039/C7CC04156J 28766595 PMC5590105

[254] Sletten ET Loka RS Yu F Nguyen HM. Glycosidase inhibition by multivalent presentation of heparan sulfate saccharides on bottlebrush polymers. Biomacromolecules. (2017) 18:3387–99. 10.1021/acs.biomac.7b01049 28846389 PMC6044434

[255] Wu L Viola CM Brzozowski AM Davies GJ. Structural characterization of human heparanase reveals insights into substrate recognition. Nat Struct Mol Biol. (2015) 22:1016. 10.1038/nsmb.3136 26575439 PMC5008439

[256] Andrgie AT Mekuria SL Addisu KD Hailemeskel BZ Hsu W-H Tsai H-C. Non-anticoagulant heparin prodrug loaded biodegradable and injectable thermoresponsive hydrogels for enhanced anti-metastasis therapy. Macromol Biosci. (2019) 19:1800409. 10.1002/mabi.201800409 30821920

[257] Wei J Long Y Guo R Liu X Tang X Rao J. Multifunctional polymeric micelle-based chemo-immunotherapy with immune checkpoint blockade for efficient treatment of orthotopic and metastatic breast cancer. Acta Pharm Sin B. (2019) 9:819–31. 10.1016/j.apsb.2019.01.018 31384541 PMC6664045

[258] Pan W Miao H-Q Xu Y-J Navarro EC Tonra JR Corcoran E. 1-[4-(1H-Benzoimidazol-2-yl)-phenyl]-3-[4-(1H-benzoimidazol-2-yl)-phenyl]-urea derivatives as small molecule heparanase inhibitors. Bioorg Med Chem Lett. (2006) 16:409–12. 10.1016/j.bmcl.2005.09.069 16246560

[259] Bathini R Fatima S Sivan SK Manga V 3D QSAR based design of novel substituted urea molecules as heparanase inhibitors. J Pharm Res. (2013) 7:754–61. 10.1016/j.jopr.2013.08.024

[260] Courtney SM Hay PA Buck RT Colville CS Phillips DJ Scopes DIC. Furanyl-1,3-thiazol-2-yl and benzoxazol-5-yl acetic acid derivatives: novel classes of heparanase inhibitor. Bioorg Med Chem Lett. (2005) 15:2295–9. 10.1016/j.bmcl.2005.03.014 15837312

[261] Kakkar S Tahlan S Lim SM Ramasamy K Mani V Shah SAA. Benzoxazole derivatives: design, synthesis and biological evaluation. BMC Chem. (2018) 12:92. 10.1186/s13065-018-0459-5 30101384 PMC6087707

[262] Papadakis M Nagel S Buchan AM Development and efficacy of NXY-059 for the treatment of acute ischemic stroke. Future Neurol. (2008) 3:229–40. 10.2217/14796708.3.3.229

[263] Dargel C Bassani-Sternberg M Hasreiter J Zani F Bockmann J-H Thiele F. T cells engineered to express a T-cell receptor specific for glypican-3 to recognize and kill hepatoma cells *in vitro* and in mice. Gastroenterology. (2015) 149:1042–52. 10.1053/j.gastro.2015.05.055 26052074

